# Structural basis for guide RNA trimming by RNase D ribonuclease in *Trypanosoma brucei*

**DOI:** 10.1093/nar/gkaa1197

**Published:** 2020-12-17

**Authors:** Yanqing Gao, Hehua Liu, Chong Zhang, Shichen Su, Yiqing Chen, Xi Chen, Yangyang Li, Zhiwei Shao, Yixi Zhang, Qiyuan Shao, Jixi Li, Zhen Huang, Jinbiao Ma, Jianhua Gan

**Affiliations:** Shanghai Public Health Clinical Center, State Key Laboratory of Genetic Engineering, Collaborative Innovation Center of Genetics and Development, Department of Physiology and Biophysics, School of Life Sciences, Fudan University, Shanghai 200438, China; Shanghai Public Health Clinical Center, State Key Laboratory of Genetic Engineering, Collaborative Innovation Center of Genetics and Development, Department of Physiology and Biophysics, School of Life Sciences, Fudan University, Shanghai 200438, China; State Key Laboratory of Genetic Engineering, Collaborative Innovation Center of Genetics and Development, Department of Biochemistry, School of Life Sciences, Fudan University, Shanghai 200438, China; College of Life Sciences, Sichuan University, Chengdu 610041, China; State Key Laboratory of Genetic Engineering, Collaborative Innovation Center of Genetics and Development, Department of Biochemistry, School of Life Sciences, Fudan University, Shanghai 200438, China; Shanghai Public Health Clinical Center, State Key Laboratory of Genetic Engineering, Collaborative Innovation Center of Genetics and Development, Department of Physiology and Biophysics, School of Life Sciences, Fudan University, Shanghai 200438, China; Shanghai Public Health Clinical Center, State Key Laboratory of Genetic Engineering, Collaborative Innovation Center of Genetics and Development, Department of Physiology and Biophysics, School of Life Sciences, Fudan University, Shanghai 200438, China; State Key Laboratory of Genetic Engineering, Collaborative Innovation Center of Genetics and Development, Department of Biochemistry, School of Life Sciences, Fudan University, Shanghai 200438, China; Shanghai Public Health Clinical Center, State Key Laboratory of Genetic Engineering, Collaborative Innovation Center of Genetics and Development, Department of Physiology and Biophysics, School of Life Sciences, Fudan University, Shanghai 200438, China; Shanghai Public Health Clinical Center, State Key Laboratory of Genetic Engineering, Collaborative Innovation Center of Genetics and Development, Department of Physiology and Biophysics, School of Life Sciences, Fudan University, Shanghai 200438, China; Shanghai Public Health Clinical Center, State Key Laboratory of Genetic Engineering, Collaborative Innovation Center of Genetics and Development, Department of Physiology and Biophysics, School of Life Sciences, Fudan University, Shanghai 200438, China; Shanghai Public Health Clinical Center, State Key Laboratory of Genetic Engineering, Collaborative Innovation Center of Genetics and Development, Department of Physiology and Biophysics, School of Life Sciences, Fudan University, Shanghai 200438, China; Shanghai Public Health Clinical Center, State Key Laboratory of Genetic Engineering, Collaborative Innovation Center of Genetics and Development, Department of Physiology and Biophysics, School of Life Sciences, Fudan University, Shanghai 200438, China; College of Life Sciences, Sichuan University, Chengdu 610041, China; State Key Laboratory of Genetic Engineering, Collaborative Innovation Center of Genetics and Development, Department of Biochemistry, School of Life Sciences, Fudan University, Shanghai 200438, China; Shanghai Public Health Clinical Center, State Key Laboratory of Genetic Engineering, Collaborative Innovation Center of Genetics and Development, Department of Physiology and Biophysics, School of Life Sciences, Fudan University, Shanghai 200438, China

## Abstract

Infection with kinetoplastid parasites, including *Trypanosoma brucei* (*T. brucei*), *Trypanosoma cruzi* (*T. cruzi*) and *Leishmania* can cause serious disease in humans. Like other kinetoplastid species, mRNAs of these disease-causing parasites must undergo posttranscriptional editing in order to be functional. mRNA editing is directed by gRNAs, a large group of small RNAs. Similar to mRNAs, gRNAs are also precisely regulated. In *T. brucei*, overexpression of RNase D ribonuclease (*Tb*RND) leads to substantial reduction in the total gRNA population and subsequent inhibition of mRNA editing. However, the mechanisms regulating gRNA binding and cleavage by *Tb*RND are not well defined. Here, we report a thorough structural study of *Tb*RND. Besides Apo- and NMP-bound structures, we also solved one *Tb*RND structure in complexed with single-stranded RNA. In combination with mutagenesis and *in vitro* cleavage assays, our structures indicated that *Tb*RND follows the conserved two-cation-assisted mechanism in catalysis. *Tb*RND is a unique RND member, as it contains a ZFD domain at its C-terminus. In addition to *T. brucei*, our studies also advanced our understanding on the potential gRNA degradation pathway in *T. cruzi*, *Leishmania*, as well for as other disease-associated parasites expressing ZFD-containing RNDs.

## INTRODUCTION

Kinetoplastids are flagellated unicellular organisms, which include many parasites responsible for serious human diseases (1). The most common of these parasitic diseases are African sleeping sickness, Chagas disease and Leishmaniasis, which are caused by infection with *T. brucei* (2–5), *T. cruzi* (6–8) and *Leishmania* (9–11), respectively. Parasitic diseases are considered a major public health issue. According to a conservative estimate, half a billion people live with the threat of trypanosomaid disease, with over 20 million infections and >100 000 annual deaths worldwide. In addition to humans, many trypanosomatids can also infect and induce serious diseases in animals (12), fish (13), and other species (14).

Although different kinetoplastids are associated with different diseases, they all share very similar cellular structure and genomic organization. Unlike other eukaryotic organisms, kinetoplastids possess an unusual genomic DNA structure, termed a kinetoplast, in their mitochondrion (15–17). The kinetoplast is composed of a few dozen maxicircles and thousands of minicircles. Maxicircles are analogous to the mitochondrial DNA of other organisms, encoding mRNAs and ribosomal RNAs, while minicircles encode for small RNAs known as guide RNAs (gRNAs). Since transcription of both maxicircles and minicircles are polycistronic (18–20), gene regulation of kinetoplastids rarely occurs at the level of RNA synthesis (21–23). In order to be translatable, most kinetoplastid mRNAs must undergo a posttranscriptional editing process that involves the insertion and deletion of uridines (24,25). As demonstrated in *T. brucei*, mRNA editing is regulated by multiple proteins; besides substrate recognition, the regulation also occurs during the initiation and procession stages of the editing (26).

gRNAs are, on average, 60 nucleotides (nt) long and function as major *trans-acting* factors in mRNA editing (22,23,27–29). All gRNAs contain three functionally distinct domains: an anchor domain at the 5′-end, a central guide domain, and a 3′-end U-tail. The anchor domain is complementary to the target mRNA and plays a fundamental role in the initiation of mRNA editing (30). The guide domain dictates the type of editing mediated by the gRNA, including the number of uridine nucleotides to be inserted or deleted. Besides the natural gRNAs, previous *in vitro* studies showed that mRNA editing could also be supported by synthetic gRNAs, which possess non-U-tails at their 3′-ends (31). Compared to the U-tails of the natural gRNAs, the non-U-tails of certain synthetic gRNAs form more Watson-Crick pairing with the target mRNAs, leading to higher editing efficiency. Although it is not preferred for particular mRNA editing *in vitro*, the U-tail is conserved in natural gRNAs. It was believed that gRNA U-tails are evolved to fit the multiple and complete editing cycles *in vivo* (31). The 3′ U-tail pairs with the purine-rich regions of target mRNAs to form a U-tail helix (32,33). In addition to the anchor helix formed between the mRNA and the gRNA anchor domain, formation of the U-tail helix contributes to the stabilization of the stem-loop structure within the gRNA guide domain. Although the pairing interactions between gRNA 3′ U-tail and the target RNA are dynamic, formation of the U-tail helix has been confirmed by both *in vitro* crosslinking assays (33,34) and structural study (35); the conformational flexibility may facilitate the structural changes of gRNA/mRNA complex during editing and allow the binding and invasion of editing proteins at the major groove (35). Deletion of the gRNA U-tail significantly lowers the *in vitro* editing efficiency of kinetoplastid mRNAs (36).

Owing to their devastating health and economic impacts, kinetoplastid parasites have been extensively studied (37–41). Editing of kinetoplastid mitochondrial mRNAs relies on the editosome, which is a multiprotein complex. Although editosomes can be divided into different subcomplexes, they all share conserved enzymes, such as ribonucleases, terminal uridylyl transferases (TUTase) and ligases (42–44). gRNAs provide the sequence information necessary for precise mRNA editing. In order to be functional, gRNAs also undergo posttranscriptional modification, especially uridylation at the 3′-end (24). This process is mediated by the mitochondrial 3′ processome, MPsome. RET1 protein, the TUTase of MPsome, catalyzes the primary uridylation (45), which stimulates hydrolytic activity of the 3′-5′ exonuclease DSS-1 (46). When the MPsome stochastically pauses at 10–12 nt from duplex region, RET1 starts the secondary uridylation.

Similar to the non-coded A-tails of mRNA, increasing evidences have shown that the U-tails at the 3′ end of small RNAs, such as siRNAs and microRNAs, also play important roles in stabilization and quality control of RNAs (47–50). In general, gRNAs accumulate in an inverse order to the edited mRNAs, suggesting that gRNA degradation is a consequence of successful mRNA editing (51). Besides *Tb*DSS-1, three other exoribonucleases are also present in mitochondrion of *T. brucei* (52–54). Of these, *Tb*REX1 and *Tb*REX2 are components of the editosome and are devoted to mRNA uridine deletion. Like *Tb*DSS-1, the final ribonuclease, *Tb*RND, also acts upon the 3′ U-tails of gRNAs. Depletion of *Tb*RND results in extended gRNA tails *in vivo*, whereas, overexpression of *Tb*RND leads to a substantial reduction in the total gRNA population and the subsequent inhibition of mRNA editing. In addition, the overexpression and RNAi-mediated knockdown assays also showed that *Tb*RND affects *T. brucei* growth in the procyclic form (55). Since the null mutant of *Tb*RND has not been reported, whether *Tb*RND is essential and involves in other biological processes in *T. brucei* need to be further investigated.

**Figure 1.**
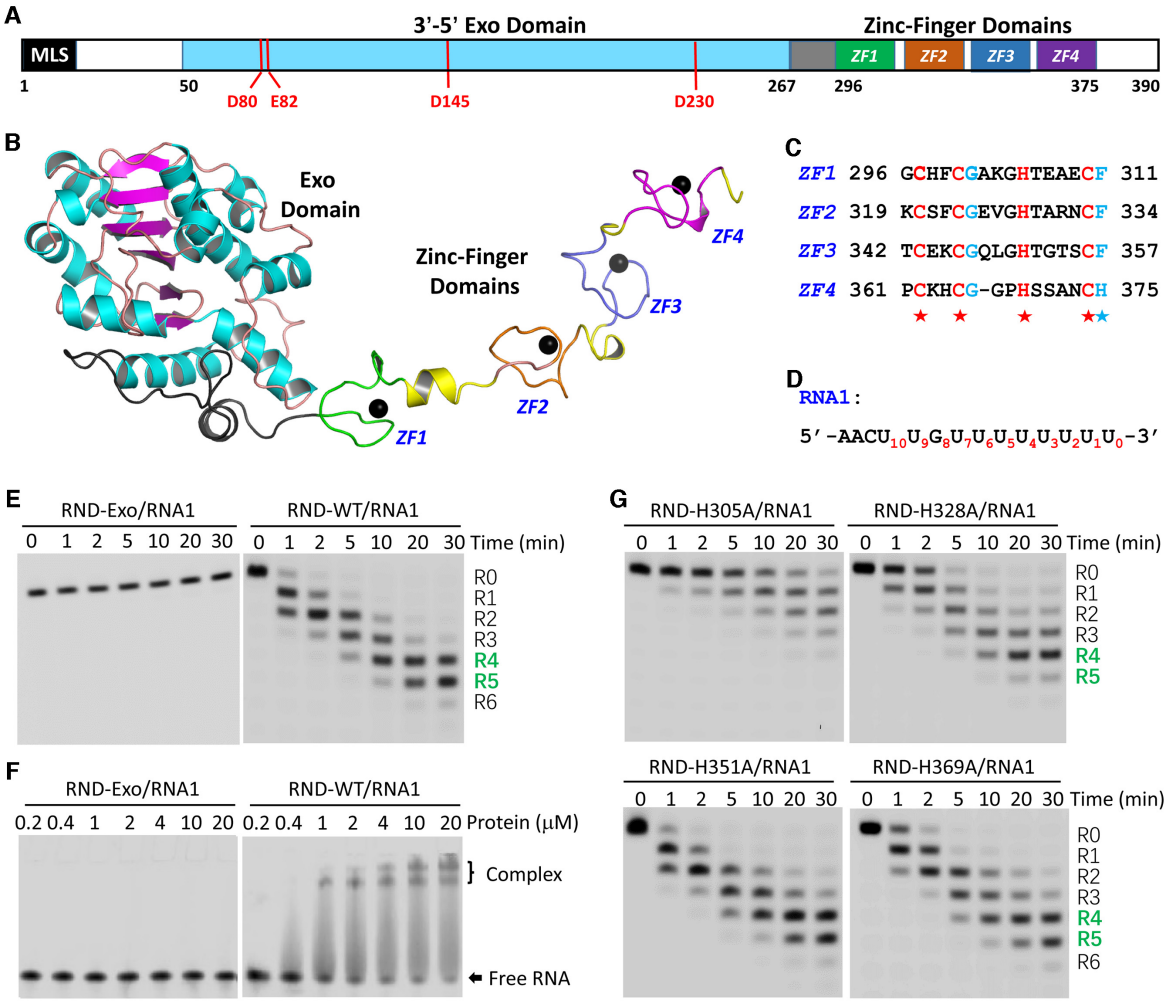
Structure and functional verification of *Tb*RND. (**A**) Domain architecture of *Tb*RND. MLS, predicted mitochondrial localization signal. (**B**) Cartoon view showing the overall structure of *Tb*RND. The Exo domain is colored in cyan and magenta for the α-helices and β-strands, respectively. The four ZF motifs, ZF1–4, are colored in green, pink, blue, and magenta, respectively. Zinc ions are shown as black spheres. (**C**) Sequence alignment of the four ZF motifs. (**D**) Sequence of RNA1 used in the *in vitro* binding and cleavage assays. (**E**) RNA1 cleavage reactions catalyzed by the Exo domain and RND-WT protein of *Tb*RND. (**F**) Comparison of RNA1 binding by the *Tb*RND Exo domain and RND-WT. (**G**) Impacts of the core Zn^2+^-coordinating Histidine mutation on RNA1 cleavage activity of *Tb*RND.

Compared to *Tb*REX1, *Tb*REX2 and *Tb*DSS-1, *Tb*RND is unique (Figure 1A), as it belongs to the RNase D (RND) group within the DEDD exoribonuclease superfamily. Like *E. coli* RNase D (*Ec*RND), *Saccharomyces cerevisiae* Rrp6 (*Sc*Rrp6), and all other RND family proteins, *Tb*RND possesses a single 3′-5′ exoribonuclease (Exo) domain. Several *Ec*RND and *Sc*Rrp6 crystal structures have been previously reported. In the native *Ec*RND structure, two metal ions were captured at the active site (56). One metal ion was bound in the active site in the *Sc*Rrp6/RNA complex structure, may be due to the mutation of the catalytic residues (57). These observations all indicated that RND family proteins follow a two-cation-assisted mechanism in catalysis, whereas a ternary complex composed of native RND protein, substrate RNA, and two coordinating cations is still unavailable. The presence of conserved 3′-5′ Exo domain suggests that *Tb*RND may share the similar mechanism in cleavage, but the overall domain architecture of *Tb*RND is significantly different from other RND members. *Ec*RND and *Sc*Rrp6 possess an HRDC (helicase and RNase D C-terminal) domain at the C-termini whereas *Tb*RND contains a predicted CCHC zinc-finger domain (ZFD) at its C-terminus. This combination of both 3′-5′ Exo and ZFD domains is very unusual and has not been observed in any reported protein structure to date. To characterize the overall folding and to unravel the basis for substrate binding and cleavage by *Tb*RND, we performed structural and functional studies. Here, we present several crystal structures, including a high-resolution structure for the RNA-bound complex, which unveil the complete fold of *Tb*RND and the interactions that facilitate RNA binding. In combination with mutagenesis and *in vitro* catalytic assays, these structures also provide detailed insights into the mechanisms for substrate binding and cleavage by *Tb*RND. In addition to *T. brucei*, our studies may also advance the general understanding of RNA metabolism in many other kinetoplastid parasites and eukaryotic organisms.

## MATERIALS AND METHODS

### Plasmid construction

The gene encoding wild-type (WT) *Tb*RND was optimized for *E. coli* expression and purchased from Yuyi Biotechnology Co., Ltd. Shanghai (Supplementary Table S1). The target fragment was amplified by PCR, digested with BamHI and XhoI, and ligated into a pET28a-Sumo vector. All truncated and mutated *Tb*RND expression constructs were created by PCR or overlap PCR using the WT *Tb*RND plasmid as template. The primers used for PCR are listed in Supplementary Table S2. The plasmid sequences were all verified by DNA sequencing. The recombinant strains were protected by 20% glycerol and stored in a −80°C freezer.

### Protein expression and purification

The recombinant plasmids were transformed into *E. coli* BL21(DE3) competent cells and cultured in Lysogeny broth (LB) medium supplemented with 50 μg/ml kanamycin at 37°C. For expression of Se-Met substituted *Tb*RND protein, the cells were cultured in M9 medium containing 50 μg/ml kanamycin and 60 mg/l Se-Met. When the OD_600_ reached 0.6–0.8, the cells were cooled down to 18°C and the protein expression induced by adding isopropyl β-d-1-thiogalacto-pyranoside (IPTG, 0.2 mM). To enhance the stability of the target protein, 0.1 mM ZnCl_2_ was included in the medium after induction. The induced cells were grown at 18°C for 16 hrs and then collected by centrifugation (4000 rpm) at 4°C for 20 min.

All proteins were purified using similar procedures. Briefly, cell pellets were resuspended with lysis buffer (20 mM Tris–HCl pH 8.0, 500 mM NaCl, 25 mM imidazole pH 8.0) and lysed under high pressure. The ensuing homogenate was clarified by centrifugation (17 000 rpm) at 4°C for 1 h. The supernatant was collected and loaded onto a HisTrap HP column (GE Healthcare). The target protein was eluted with 20 mM Tris–HCl pH 8.0, 500 mM NaCl, 500 mM imidazole pH 8.0. The protein was treated with Ulp1 protease at 4°C for 3 h to remove the His-Sumo tag, diluted with 20 mM Tris–HCl pH 8.0, 100 mM NaCl and loaded onto a HiTrap Heparin HP column (GE Healthcare). The target protein was eluted with buffer composed of 20 mM Tris–HCl pH 8.0 and 300 mM NaCl. The eluted protein was further purified by gel filtration using a HiLoad 16/60 Superdex 75 column in gel-filtration buffer (20 mM Tris–HCl pH 8.0, 200 mM NaCl, 2 mM DTT).

### Crystallization and x-ray diffraction data collection

The initial crystallization conditions were all identified using the Gryphon crystallization robot system and commercial crystallization kits, using the sitting-drop vapor diffusion method at 16°C. Crystallization was optimized by the hanging-drop vapor diffusion method. All nucleic acids used for crystallization were synthesized and purified in the laboratory. The *Tb*RND ΔZF_3–4/RNA-12U complex was prepared by mixing 10 mM CaCl_2_, *Tb*RND ΔZF_3–4 (residues 41–341) with RNA-12U (5′-UUUUUUUUUUUU-3′). The molar ratio between ΔZF_3–4 and RNA-12U is 1:1.2. The crystallization condition for the ΔZF_3–4/RNA-12U complex was 0.2 M Sodium acetate trihydrate, 20% PEG3350, while that for the Se-Met substituted Apo-form of *Tb*RND was 0.1 M Bis–Tris pH 5.5 and 30% PEG3350. The NMP-bound *Tb*RND crystals were obtained by soaking the Apo-*Tb*RND crystals in mother liquor supplemented with 10 mM MnCl_2_ and 10 mM NMP for 10min.

All crystals were cryo-protected in reservoir solution supplemented with 25% (v/v) glycerol and snap-frozen in liquid nitrogen. The X-ray diffraction data were collected on beamlines BL17U, BL18U, and BL19U at the Shanghai Synchrotron Radiation Facility (SSRF). The HKL3000 program package (58) was used to process the data. The data collection and processing statistics are summarized in Supplementary Table S3.

### Structure determination and refinement

The Apo-form Se-Met substituted *Tb*RND structure was solved by the single-wavelength anomalous diffraction (SAD) method (59) with the Autosol program embedded in the Phenix suit (60). The initial model was built using the Autobuilt program and then refined against the diffraction data using the Refmac5 program of the CCP4 suite (61). The 2*F*_o_ – *F*_c_ and *F*_o_ – *F*_c_ electron density maps were regularly calculated and used as guide for the building of the missing amino acids using COOT (62). The NMP-bound or RNA-complexed *Tb*RND structures were solved by molecular replacement using the apo-*Tb*RND structure as the search model with the phaser program of the CCP4 suite. Nucleic acids, ions, water, and other molecules were all built manually using COOT. The complex structures were also refined using the Refmac5 program of the CCP4 suite. The structural refinement statistics were summarized in Supplementary Table S3.

### Substrate binding assays

Electrophoretic mobility shift assays (EMSA) were used to analyze the RNA binding ability of *Tb*RND proteins. The 10-μl reaction mixture was composed of protein, 0.2 μM FAM-labeled substrate, 20 mM Tris–HCl pH 8.0, 200 mM NaCl, and 2 mM DTT. The mixture was incubated on ice for 1 h before adding 2 μl of loading buffer (12% Ficoll 400 and 5% glycerol). The samples were then loaded onto a pre-cooled 6% native polyacrylamide gel. The gels were run at 100 V for 40 min at 4°C in 0.5× TBE buffer and imaged using a Typhoon FLA 9000. The intensities of the bands were quantified by ImageQuantTL.

### *In vitro* cleavage assays

FAM-labeled substrates and cognate mRNA (Supplementary Table S4) were purchased from the TAKARA company. The reactions were carried out in a 10-μl system containing WT or mutated *Tb*RND proteins, 0.4 μM substrates, 0.4 μM mRNA (if present), 20 mM Tris–HCl pH 8.0, 50 mM KCl, 1 mM EDTA, 10% glycerol, 1 mM DTT, 5 mM MgCl_2_. The protein concentration was 0.1 μM for RND-WT and related mutants (residues 41–390), whereas it was increased to 1.0 μM for RND-Exo (residues 41–296). The reaction mixtures were incubated at 24°C for different lengths of time, after which they were terminated by adding 10 μl termination buffer (95% formamide, 25 mM EDTA) and heating at 95°C for 5 min. The samples were loaded onto pre-warmed 18% polyacrylamide 7-M urea gels and run at 10 W for 2 h. The gels were scanned using a Typhoon FLA 9000. The intensities of the bands were quantified by ImageQuantTL.

## RESULTS

### Overall structure of *Tb*RND

The *Tb*RND enzyme is encoded by the Tb09.211.3670 gene and is 390 amino acids in length (Figure 1A). A previous study suggested that full-length *Tb*RND was not very stable when expressed in *E. coli*. In order to improve protein stability sufficiently for crystallographic analysis, we designed and screened various constructs to determine that removal of the N terminal mitochondrial localization signal (MLS) significantly improved the stability of *Tb*RND. Using truncated proteins, we solved several *Tb*RND crystal structures, including an Apo-form, four NMP-bound forms, and one in complex with RNA-12U (Supplementary Table S3). The GMP-bound (G-form, 2.25Å) crystals were formed by soaking GMP into Apo crystals grown with the wild-type (WT) *Tb*RND lacking the N-terminal 40 residues (hereafter referred to as RND-WT) and unveiled the most complete structural information for *Tb*RND.

This G-form crystal belonged to the *P*2_1_2_1_2_1_ space group and contained one *Tb*RND molecule per asymmetric unit. As depicted in Figure 1B, the *Tb*RND Exo domain is composed of 218 residues (amino acids 50–267) arranged in an α/β fold. All but one of the six β-strands are parallel, forming a single flat β-sheet flanked by α-helices on both sides. The Exo and ZFD domains are connected by a linker (amino acids 268–295) composed of two loops bisected by a short α-helix. The ZFD domain (amino acids 296–375) contains four Zinc-finger motifs (ZF1–4) that adopt an extended conformation (Figure 1B). All the ZFs belong to the CCHC-type; where they all possess one aromatic residue, either Phe or His, following the last Cys (Figure 1C). Compared to ZF1–3, the loop connecting the central Zinc-coordinating Cys and His residues for ZF4 is one amino acid shorter. However, the overall folds and Zinc-coordinations of the four ZFs are very similar (Supplementary Figure S1).

Like the G-form structure, the ZFD also adopts an extended conformation in the Apo-form structure. Structural analysis suggested that the extended ZFD conformations are mainly stabilized by crystal packing. In the G-form structure, ZF4 forms several hydrogen bonding (H-bond) interactions with the Exo domain of symmetry-related molecule, which stabilized ZFD from the distal end (Supplementary Figure S2A-B). Although ZF3 and ZF4 are also present in the Apo-form structure, they are disordered, suggesting that they don’t form stable interactions with surrounding molecules (Supplementary Figure S2C). In contrast, ZF2 is well defined in both structures, due to its extensive interactions with symmetry-related molecules (Supplementary Figure S2D, E).

### ZFD enhances RNA binding and cleavage activities of *Tb*RND

Guided by the crystal structure, we constructed and purified a *Tb*RND variant (amino acids 41–296) with the ZFD domain deleted, hereafter referred to as RND-Exo. To investigate the functions of the individual domains, we performed *in vitro* cleavage assays. As expected RND-WT could efficiently cleave RNA-12U (5′-FAM-UUUUUUUUUUUU-3′) such that after a reaction time of 1 min, 57.51% of the substrate was cleaved (Supplementary Figure S3A). Only a trace amount of substrate was observed after 30 min, with the main products being five or six nucleotides (nt) shorter than the substrate strand. Compared to RND-WT, cleavage efficiency of the RNA-12U by RND-Exo is much lower (Supplementary Figure S3B). Even with a 10-fold higher concentration (1.0 μM), RND-Exo can only cleave 33.97% of RNA-12U after 30 min. When the reaction time was extended to 90 min, 19.68% of the substrate remained intact. Both RND-WT and RND-Exo had extremely low cleavage activities toward RNA-12A and RNA-12C RNAs (Supplementary Figure S3). Consistent with the previous study (55), these observations suggested that *Tb*RND has a strong preference for U-rich RNAs.

In addition to RNA-12U, we also performed *in vitro* cleavage assay using another U-rich RNA, RNA1 (Figures 1D, E, Supplementary Table S5). RNA1 (5′-FAM-AACUUGUUUUUUUU-3′) was designed to mimic the 3′-end of gRNAs, the natural substrate for *Tb*RND. Similar to RNA-12U, RNA1 was cleaved by RND-WT at a concentration of 0.1 μM, with the overall cleavage efficiencies being similar. At a reaction time of 30 min, the major RNA1 cleavage products had 4 or 5 nucleotides removed from the 3′-end with a total yield of 88.38%. However, at the same concentration, RND-Exo could only generate trace amounts of product, which were 1-nt shorter than RNA1.

The above cleavage assay results clearly indicated that the ZFD domain enhances the RNA cleavage activity of *Tb*RND. To better understand the function of the *Tb*RND ZFD, we carried out *in vitro* binding assays using RNA1 (0.2 μM) by EMSA. As depicted in Figure 1F, the binding affinity of RND-Exo for RNA1 is very low. Even under conditions with 100-fold molar excess of RND-Exo (20 uM), no obvious band-shift was observed for RNA1. In contrast, RND-WT displayed strong binding affinity for RNA1. 25.24% of the RNA1 was shifted in the presence of RND-WT at a concentration of 0.4 μM. Increasing the concentration of RND-WT to 4.0 μM resulted in a shift of 85.30% of RNA1, and 92.36% RNA1 was shifted in the presence of 20 μM RND-WT.

The binding assay results suggested that the ZFD domain plays a critical role in RNA binding, which may in turn enhance the RNA cleavage activity of *Tb*RND. To investigate the contribution of each individual ZF motif, we constructed four *Tb*RND mutants, H305A, H328A, H351A and H369A, in which the core Zn^2+^-coordinating Histidine residues of ZF1–4 was sequentially substituted by Alanine. Similar to RND-WT, we also performed *in vitro* cleavage assays using RNA1 and the four mutant proteins (Figure 1G, Supplementary Table S5). In contrast to RND-WT, the RNA1 cleavage activity of H305A mutant is very weak. At a reaction time of 30 min, there still had 13.09% intact RNA1 left and the major products only had 1 or 2 nucleotides removed from the 3′ end. Compared to RND-WT, the RNA1 cleavage activity of H328A mutant is also weaker. At a reaction time of 30 min, it mainly produced a product that had four nucleotides removed with a yield of 45.56%. Different from H305A and H328A, the H351A and H369A mutants showed similar RNA1 cleavage activity as that of RND-WT at a reaction time of 30 min.

### Structural basis for RNA binding by *Tb*RND ZFD

**Figure 2.**
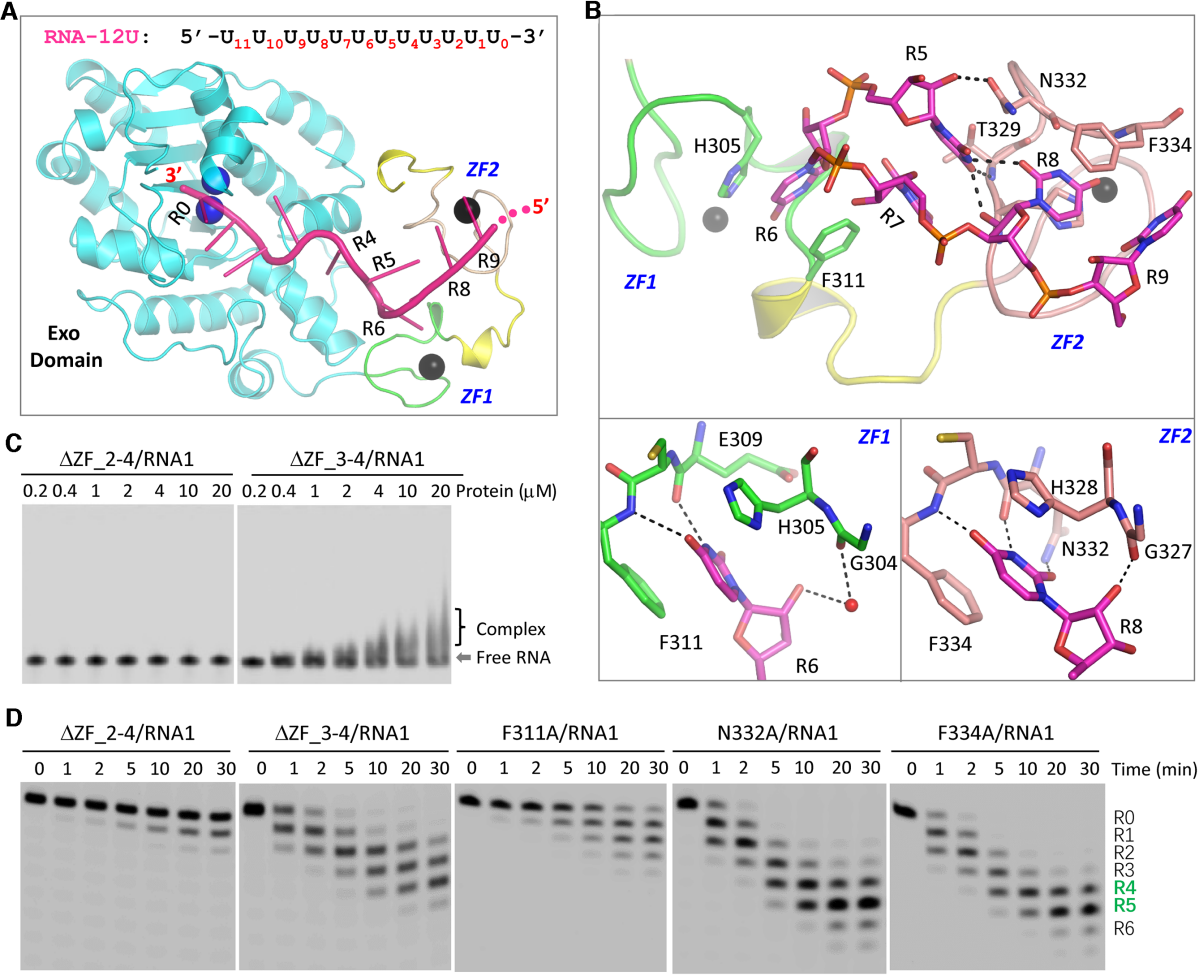
RNA recognition by the ZFD domain of *Tb*RND. (**A**) Cartoon representation showing the overall fold of the ΔZF_3–4/RNA-12U complex. The sequence and R# notations of RNA-12U are shown at the top. (**B**) The interactions between RNA-12U and the ZF1 and ZF2 motifs of *Tb*RND. (**C**) *In vitro* RNA binding by truncated versions of *Tb*RND removing the various ZF motifs. (**D**) Impact of mutation of ZFD RNA-interacting residues on RNA cleavage by *Tb*RND. In panels A and B, the C-atoms of the Exo domain, ZF1, ZF2 and RNA-12U are colored in cyan, green, pink, and magenta, respectively. Zinc ions, Ca^2+^ ions, and water molecules are shown as spheres in black, blue, and red, respectively. H-bond interactions are indicated by black dashed lines.

To unravel the mechanisms underlying RNA binding by *Tb*RND, we performed extensive co-crystallization trails. Although no crystals grew for the mixture of RNA and RND-WT, we successfully solved the structure for a complex of RNA-12U bound to the *Tb*RND ΔZF_3–4 mutant (amino acids 41–341), a truncation in which ZF3 and ZF4 were deleted. The structure was termed ΔZF_3–4/RNA-12U and refined to high resolution (1.77 Å, Supplementary Table S3). Each molecule of *Tb*RND ΔZF_3–4 binds to one RNA-12U molecule (Figure 2A). Of the 12 nucleotides in RNA-12U, 10 were well-ordered and labeled R0 to R9 from the 3′ end (Supplementary Figure S4). The Zinc fingers, ZF1 and ZF2, of *Tb*RND mainly interact with the nucleotides at the R5–R9 region (Figure 2B). R5 forms two H-bond interactions with ZF2, including one between its ribose 2′-OH group and the side chain OD1 atom of Asn332 and the other between the nucleobase O4 atom and the main chain N atom of Thr329. The conformation of R5 is further stabilized by its H-bond interactions with R8 and stacking interactions with R7, which resides at the interface between ZF1 and ZF2.

The nucleobase of R6 inserts into a shallow groove in ZF1 (Figure 2B, bottom-left panel), such that the N3 and O4 atoms form stable H-bond interactions, of around 2.8 Å distance, with the main chain O and N atoms of Glu309 and Phe311, respectively. The ribose 2′-OH group also forms a water-mediated H-bond interaction with the main chain O atom of Gly304. The groove in which the nucleobase of R6 is inserted, is bounded on either side by Phe311 and His305, thus forming extensive hydrophobic interactions that stabilize this interaction.

R8 interacts with ZF2 (Figure 2B, bottom-right panel), in a similar manner to that observed for R6 and ZF1, including hydrophobic stacking with aromatic residues, His328 and Phe334, and by direct H-bond interactions with the main chain atoms of Asn332 and Phe334. This interaction is further stabilized by a H-bond between the R8 nucleobase and the side chain of Asn332. The ribose of R8 is also involved in this interaction. However, instead of a water-mediated H-bond, the 2′-OH group of R8 directly H-bonds to the main chain O atom of Gly327. Unlike R5, R6 and R8, the nucleobases of R7 and R9 do not form direct H-bonds, with water-mediated H-bonds observed with either ZF1 or ZF2, further stabilizing the conformation of RNA-12U in the bound complex.

To further confirm the functional importance of ZF1 and ZF2, we constructed a *Tb*RND ΔZF_2–4 mutant (amino acids 41–317), in which ZF2–4 were all deleted, for use in *in vitro* RNA binding and cleavage assays (Figures 2C-D, Supplementary Table S5). Similar to RND-Exo, ΔZF_2–4 has very weak RNA1-binding affinity. Although it is weaker than RND-WT, ΔZF_3–4 could shift RNA1 at a concentrations at or above1.0 μM. The RNA1 cleavage activities of ΔZF_2–4 and ΔZF_3–4 are higher than that of RND-Exo. At a reaction time of 30 min, ΔZF_3–4 could produce 44.50% product that had 4 nucleotides removed from the 3′ end. Compared to H351A and H369A (Figure 1G, lower panel), the RNA1 cleavage activity of ΔZF_3–4 is weaker. Taken together, these observations suggested that all four ZF motifs are important for the cleavage activity of *Tb*RND. The functions of ZNF3 and ZNF4 motifs might be redundant with each other. Blocking the interactions between RNA and single ZNF motif has no strong impact. However, if the interactions between RNA and both ZNF3 and ZNF4 are blocked, it will lower the cleavage activity of *Tb*RND.

Based on the ΔZF_3–4/RNA-12U complex structure, we constructed three single-point mutants of *Tb*RND (F311A, N322A, and F334A) and performed *in vitro* cleavage assays (Supplementary Table S5). Substitution of ZF1 residue Phe311 by Ala (for F311A) significantly lowered the RNA1 cleavage activity of *Tb*RND (Figure 2D). After 30 minutes of cleavage, 12.21% of the substrate remained uncleaved. Compared to RND-WT (Figure 1E, right panel), F311A-generated products had much fewer nucleotides removed from the 3′-ends, may be due to the extensive interactions between the RNA and ZF2. As indicated by the similar panels and total yields of the products (Supplementary Table S5), the single Ala substitution of Asn322 (for N322A) or Phe334 (F334A) had no significant impact on RNA1 cleavage by *Tb*RND.

### Interactions between RNA and *Tb*RND Exo domain

**Figure 3.**
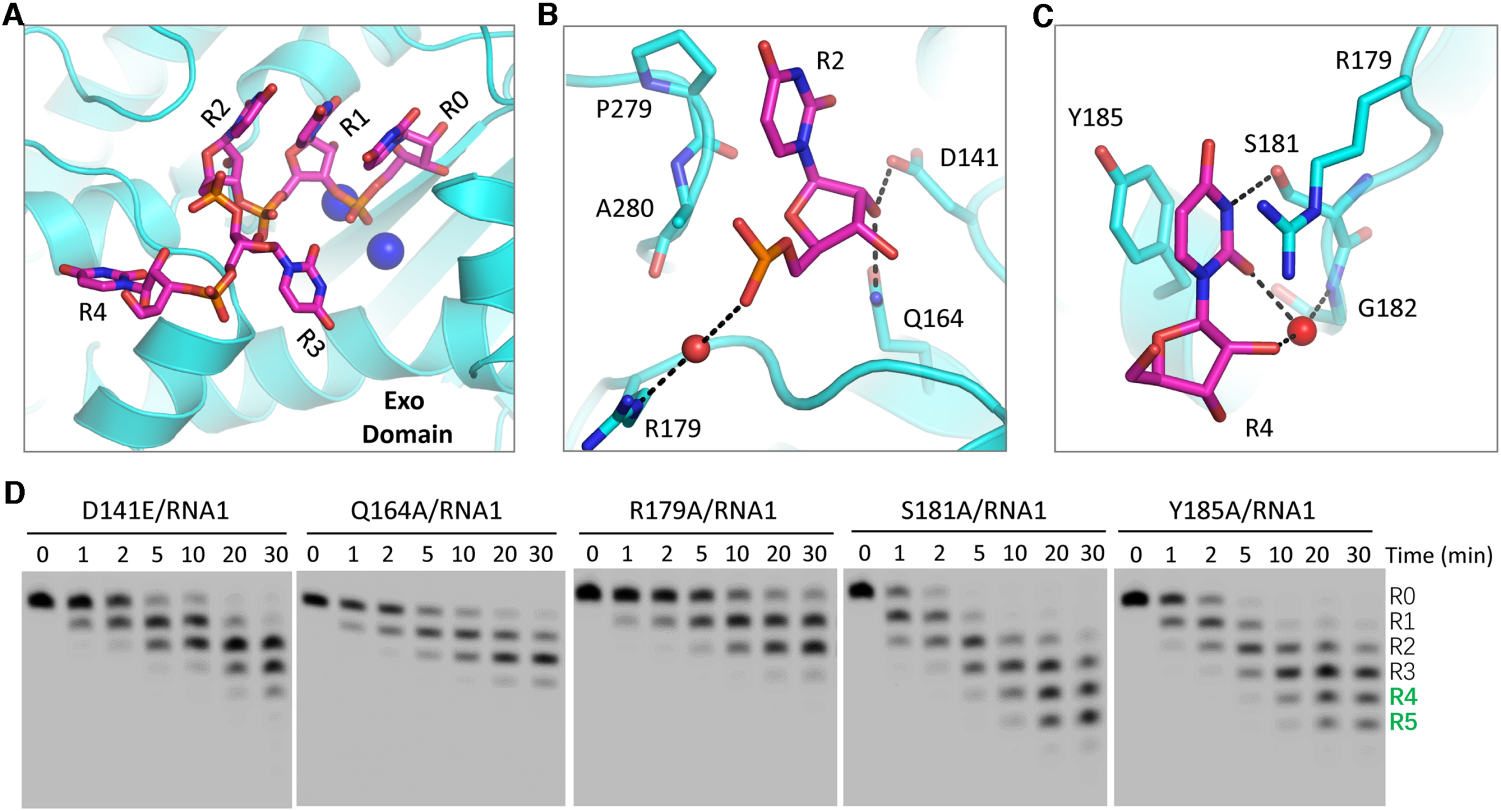
Interactions between RNA and the Exo domain of *Tb*RND. (**A**) Stick presentation showing the nucleotides bound in the Exo domain of *Tb*RND. The Exo domain and Ca^2+^ ions are shown as cyan cartoon and blue spheres, respectively. (**B**, **C**) The binding of the R2- and R4-site nucleotides by the Exo domain of *Tb*RND. Water molecules are shown as red spheres, H-bond interactions are indicated by black dashed lines. (**D**) Impacts of mutation of the Exo RNA-interacting residues on RNA cleavage by *Tb*RND.

The Exo domain of *Tb*RND is responsible for RNA degradation (Supplementary Figure S3B). Although RND-Exo alone does not show clear RNA1-binding affinity *in vitro*, the Exo domain forms extensive interactions with the RNA in the ΔZF_3–4/RNA-12U complex structure, mainly recognizing nucleotides R0-R4 located at the 3′-end of RNA-12U. Nucleotides R0, R1 and R2 are bound inside an open pocket with the nucleobases stacked against each other to form an A-form-like conformation (Figure 3A). The R2 nucleobase packs against Pro279, forming a weak hydrophobic interaction with the CB atom (Figure 3B). The 2′-OH group of the R2 ribose ring forms two H-bonds, one each with the OD2 atom of Asp141 and the NE2 atom of Gln164, respectively. The backbone phosphate group of R2 interacts with the side chain of Arg179 through a water mediated H-bond.

The nucleobases of R3 and R4 are rotated in opposite directions such that they are perpendicular to those of R0, R1 and R2, but point in different directions (Figure 3A). No base-specific interaction is observed between R3 and *Tb*RND. In contrast, R4 forms extensive interactions with *Tb*RND (Figure 3C), with the nucleobase being flanked by Arg179 on one side and Tyr185 on the other side. The N3 atom of the R4 nucleobase forms an H-bond interaction with the OG atom of Ser181. The nucleobase O2 atom and ribose 2′-OH group of R4 both form indirect H-bonds, via a water molecule, with the main chain N atom of Gly182.

Directed by the structural observations described above, we designed and constructed several Ala-substituted mutants of *Tb*RND and performed *in vitro* cleavage assays (Figure 3D). Q164A, R179A, S181A and Y185A mutants expressed in *E. coli* as soluble stable proteins, whereas, D141A was expressed into inclusion bodies. To investigate the impact of Asp141, we alternatively constructed and purified a D141E mutant, in which Asp141 was substituted with a Glu residue. Compared to RND-WT (Figure 1E, right panel), the RNA1 cleavage activity of the D141E mutant was significantly lower. At a reaction time of 30 min, D141E only produced 12.31% and 1.09% products with 4 and 5 nucleotides removed for the 3′ end, respectively. More dramatic reductions were observed for the Q164A and R179A mutants. No products with either four or five nucleotides removed from the 3′ end were generated, the main products are only 1 or 2-nt shorter than the substrate. Although not as significant as for the mutations in the previous three residues, Ala-substitution of Ser181 (for S181A) and Tyr185 (for Y185A) also caused weak reduction in the RNA1 cleavage activity of *Tb*RND, as indicated by the low percentage of products that had four or five nucleotides removed from the 3′ end (Supplementary Table S5).

### Two-cation-assisted mechanism of *Tb*RND

**Figure 4.**
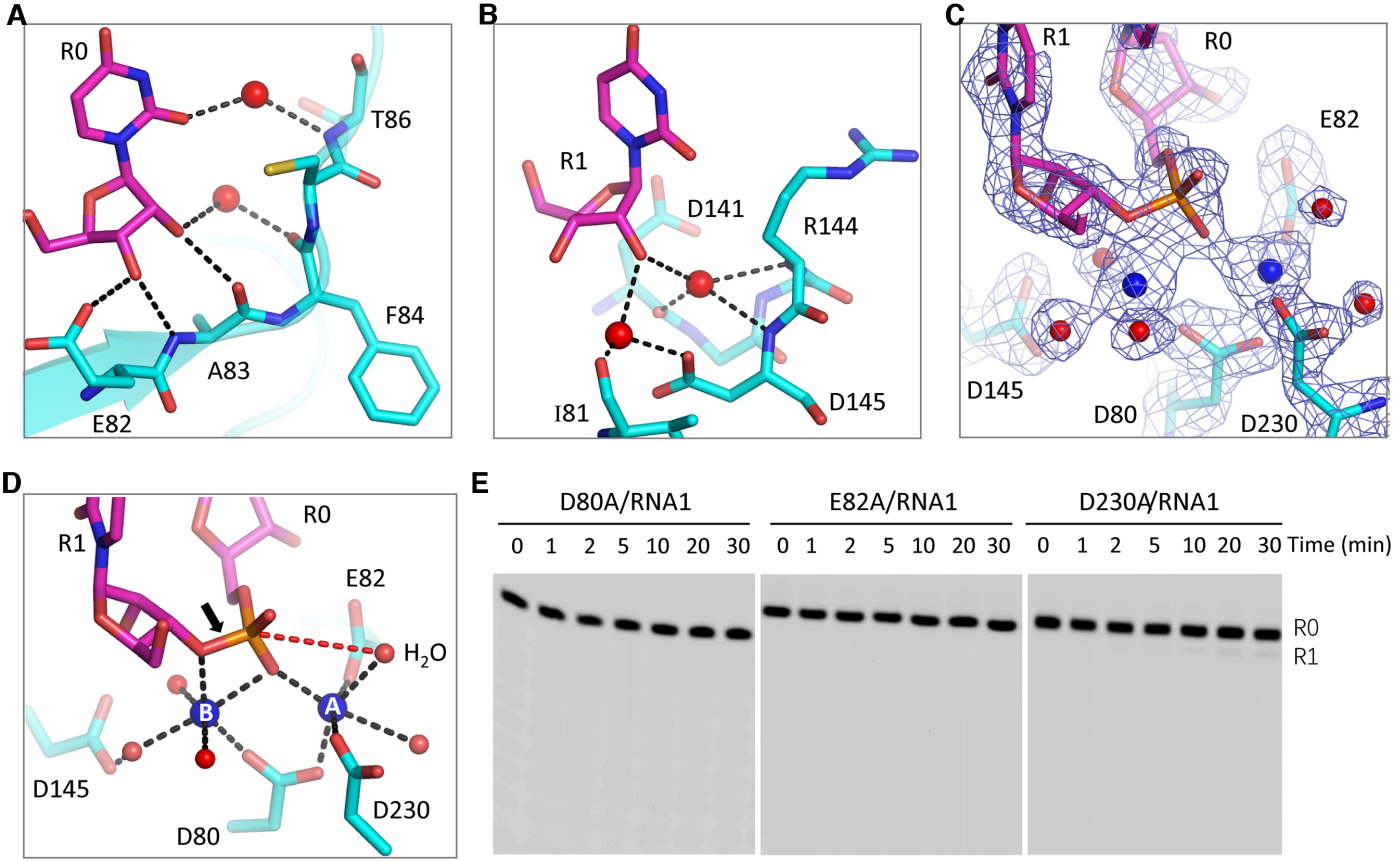
The catalytic mechanism of *Tb*RND. (**A**, **B**) Cartoon depiction of the interactions of the cleavage site nucleotides, R0 and R1, with the protein. (**C**) 2F_o_-F_c_ electron density map of the nucleotides, Ca^2+^ ions, water, and the active site residues. The maps are contoured at 1.5 σ. (**D**) The detailed coordination of the Ca^2+^ ions and the two-cation-assisted mechanism of *Tb*RND. (**E**) Impact of catalytic site residue mutation on RNA cleavage by *Tb*RND. In panels A–D, the C-atoms of *Tb*RND and RNA are colored in cyan and magenta, respectively. Ca^2+^ ions and water molecules are shown as blue and red spheres, respectively.

During *Tb*RND degradation of RNA from the 3′-end, the P-O bond between R0 and R1 is broken during catalysis. As observed in the ΔZF_3–4/RNA-12U complex structure, the 3′ end R0 nucleotide forms extensive interactions with *Tb*RND (Figure 4A). The 3′-OH group on the ribose of R0 forms two stable H-bonds, with the side chain OE1 atom of Glu82 and the main chain N atom of Ala83. The ribose 2′-OH group of R0 interacts with the main chain O atom of Ala83. As indicated by the short distances (∼2.8 Å), these H-bond interactions are all very stable. R0 also forms water-mediated H-bond interactions with Phe84 and Thr86 of *Tb*RND, with the 2′-OH group and the nucleobase O2 atoms, respectively. R1 also interacts with *Tb*RND, mainly through H-bonds that are mediated by water molecules (Figure 4B).

The enzymatic activity of DEDD family nucleases is chemically facilitated by divalent cations coordinated in the active site, commonly Mg^2+^. As exhibited by the structures of many nucleases, including RNase H (63), RNase III (64), and C3PO (65), Ca^2+^ can mimic Mg^2+^ in coordination but will not catalyze the cleavage reaction. The crystals of the ΔZF_3–4/RNA-12U complex were grown in the presence of 10 mM CaCl_2_. In the structure, two well-defined Ca^2+^ ions were captured in the active site (Figure 3C) in a hexacoordinated octahedral-like geometry (Figure 3D). The A-site ion is coordinated by the side chains of the Asp80, Glu82, and Asp230 residues, as well as the OP1 atom of R0 and two water molecules. The B-site ion is coordinated by the side chain of Asp80, the OP1 atom of R0, and three water molecules. In addition, the B-site ion is also coordinated by the O3′ atom of R1. Asp80, Glu82, Asp145 and Asp230 correspond to the four residues conserved in the DEDD family nucleases. Although Asp145 does not form a direct interaction with either of the active site cations, it does form an H-bond with a cation-coordinating water molecule.

The above structural observations suggested that *Tb*RND use the two-cation-assisted mechanism for RNA cleavage, which is also followed by RNase H (63), RNase III (64), and many other nucleases. The A-site cation will activate the nucleophile water molecule via deprotonation, which will then attack the phosphorus atom of the R0 nucleotide. In addition to assembly of the catalytic form complex, the B-site cation can also facilitate the P–O bond breakage by neutralizing the negative charge that develops on the 3′ oxygen atom of the R1 nucleotide. To verify the catalytic mechanism, we constructed three *Tb*RND mutants of the cation-coordinating residues. As shown by *in vitro* cleavage assays (Figure 4E), Ala substitution of either Asp80 (for D80A) or Glu82 (for E82A) completely abolished the RNA cleavage activity of *Tb*RND. Compared to that of RND-WT, the RNA cleavage activity of the D230A mutant was significantly lower such that only trace amounts of the products were generated after cleavage for 30 min. Taken together, these observations indicated that Asp80, Glu82 and Asp230 are all critical for RNA degradation by *Tb*RND.

### Nucleotide binding and uridine preference of *Tb*RND ZFD

**Figure 5.**
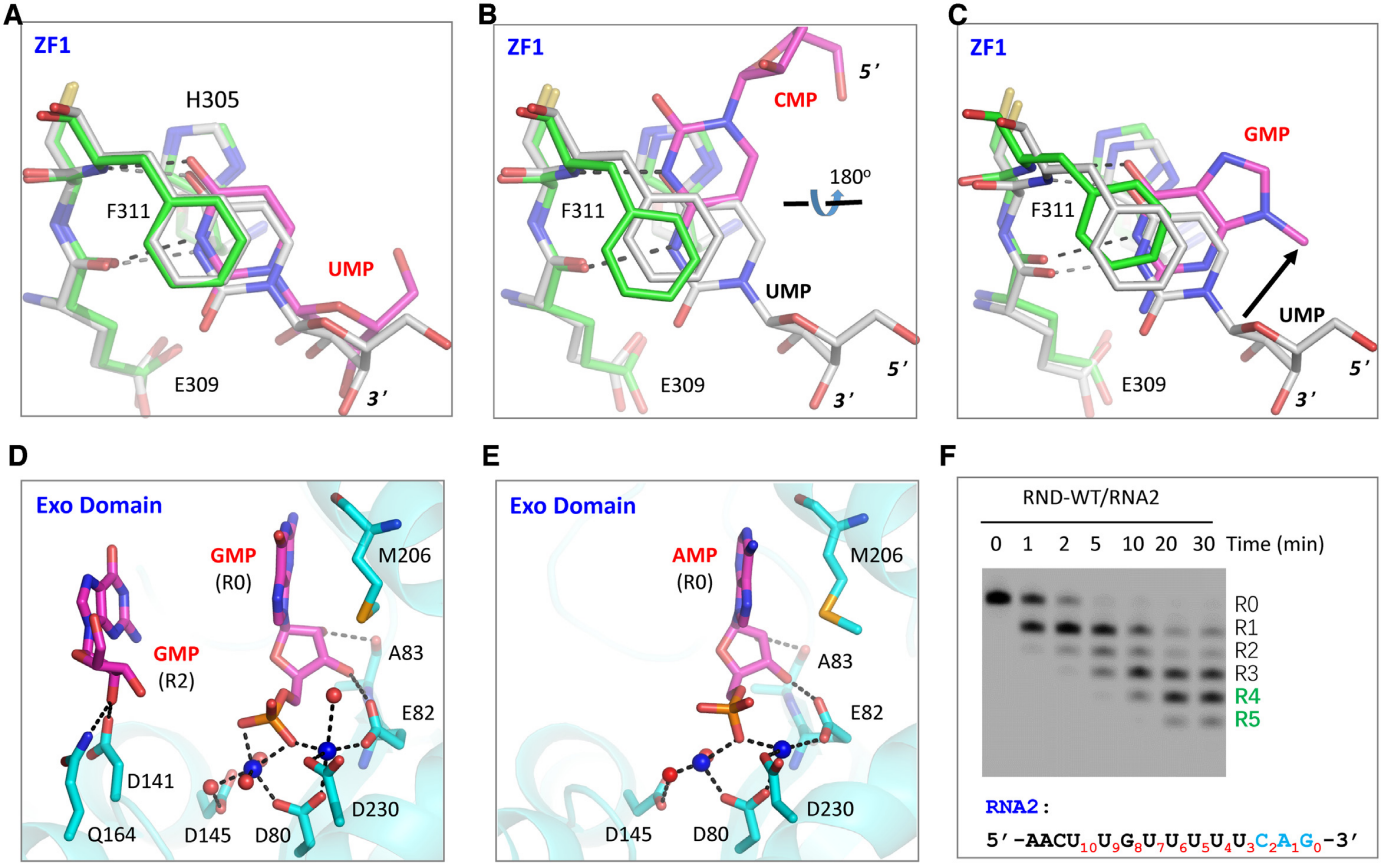
NMP binding and selection by *Tb*RND. (**A**) Superposition of UMP bound in the ZF1 groove of *Tb*RND. (**B, C**) Conformational comparison of CMP and GMP with UMP bound in the ZF1 groove of *Tb*RND. (**D**) Stick-and-sphere view showing GMPs bound in the *Tb*RND Exo domain of the GMP-bound structure. (**E**) AMP bound in *Tb*RND Exo domain in the AMP-bound structure. (**F**) *In vitro* RNA2 cleavage by RND-WT protein. In panels A–C, the C-atoms are colored in grey for both UMP and ZF1 residues of the ΔZF_3–4/RNA-12U complex. For the NMP-bound structures, the C-atoms are colored in green and magenta for ZF1 and NMP, respectively. In panels D and E, Mn^2+^ ions and the coordinating water molecules are shown as blue and red spheres, respectively.

Both this and previous studies have shown that *Tb*RND has a strong preference for U-rich sequences (Supplementary Figure S3A). To better understand the RNA sequence preference of *Tb*RND, we solved structures for all four NMP-bound complexes of *Tb*RND. Like the G-form structure, used to define the overall structure described above, all the NMP-bound crystals were formed from soaks of the Apo RND-WT crystals. However, as indicated by the large cell parameter differences (Supplementary Table S3), the packing of the RND-WT was different in the crystal lattices of these soaked crystals, leading to disordering of ZF3 and ZF4 in all of the other NMP-bound structures, when compared with the G-form structure. In the UMP-bound (U-form) structure, one well-defined UMP molecule was captured in the groove of ZF1 (Supplementary Figure S5A, B). Structural superposition (Figure 5A) showed that binding of UMP is identical to that of the R6 UMP in the ΔZF_3–4/RNA-12U complex, including the H-bond (with Glu309 and Phe311) and stacking (with His305 and Phe311) interactions. In the CMP-bound (C-form) structure, one CMP nucleotide was bound in ZF1 groove (Supplementary Figure S5C, D). Unlike for UMP, CMP forms two H-bond interactions with ZF1, including one each respectively between the N3 and N4 atoms of the nucleobase and the main chain N atom of Phe311 and the main chain O atom of Glu309. Compared to R6 in the ΔZF_3–4/RNA-12U complex, the nucleobase of CMP was rotated 180° around the C4-C5 bond of the nucleobase, disrupting the stacking interaction with Phe311 (Figure 5B).

In the G-form structure, one GMP molecule was captured within the ZF1 groove (Supplementary Figure S5E-F), where the N1 and O6 atoms of GMP form weak H-bond interactions, as defined by the relatively long distances (3.0 Å), with the main chain O and N atoms of Glu309 and Phe311, respectively. Like CMP, the nucleobase of GMP does not form a stable stacking interaction with Phe311, consistent with the high B-factors (>70 Å^2^) and very weak electron density for Phe311. Due to this weak binding and associated disorder, the ribose of GMP was not observed in the G-form structure. However, structural superposition suggested that the GMP ribose points in a different direction from that of R6 UMP in the ΔZF_3–4/RNA-12U complex (Figure 5C). Unlike the other NMP-bound structures, no AMP was found in the ZF1 groove of the structures for the ATP soaked crystals. Taken together, these observations indicated that the ZF1 groove has a preference for uridines and the significant orientational change observed for the CMP bound to ZF1 (Figure 5B), suggest that this preference is driven not only by the size and shape of the nucleobase, but also the global conformational landscape of the RNA strand.

One GMP molecule was also observed in the ZF2 groove of the G-form structure (Supplementary Figure S6A-B). This interaction was largely dependent on the presence of a Mn^2+^ ion, from the soaking condition, observed coordinated to a negatively charged Glu325. No negative residues are located at the corresponding position in the other *Tb*RND ZFs. In the crystal lattice of the U-form structure, the ZF2 groove is very close to a symmetry-related molecule, which may prevent UMP from binding to the ZF2 groove, through steric occlusion. Like ZF2, ZF3 and ZF4 also share conserved folds with ZF1 (Supplementary Figure S1). Although it was not observed in our structures, the structural similarities suggested that ZF2–4 may also have certain preference for uridine residues.

### 3′-End nucleotide tolerance of *Tb*RND Exo

As demonstrated by the ΔZF_3–4/RNA-12U complex structure, the 3′-end nucleotides R0, R1 and R2 of RNA substrate do not form base-specific interactions. R0, R1, and R2 interact with *Tb*RND Exo, but these interactions are mainly mediated by their ribose or phosphate groups (Figures 3B and 4A, B). During soaking experiments, no UMP was found inside the open pocket of *Tb*RND Exo, whereas we observed two GMP molecules in the G-form structure (Figure 5D, Supplementary Figure S6C), located at the R0 and R2 sites, respectively. No AMP was bound in the R2 site, but one AMP was captured at the R0 site in the A-form structure (Figure 5E, Supplementary Figure S6D). Structural analysis showed that the binding modes of GMP and AMP to the R0-site are very similar to that observed for the R0 UMP in the ΔZF_3–4/RNA-12U complex. Likewise, the R2-site bound GMP and CMP, in the G-form and C-form structures, respectively, had similar binding modes to that of R2-site UMP in the ΔZF_3–4/RNA-12U complex.

Although *in vitro* cleavage assays suggested that *Tb*RND had very poor poly(A) or poly(C) cleavage activities (Supplementary Figure S3A), both our NMP-bound and RNA-complexed structures suggested that *Tb*RND has no strong sequence preference for the 3′-end nucleotides of the RNA substrates. To further confirm this hypothesis, we performed an *in vitro* cleavage assay using RNA2 (5′-FAM-AACUUGUUUUUCAG-3′), which is identical to RNA1 at the 5′-end, with a CAG substituted for the RNA1 UUU at the 3′-end. As depicted in Figure 5F, *Tb*RND can efficiently degrade RNA2, such that after 5 min of reaction time, almost all the RNA2 was cleaved. Taken together, these observations indicated that *Tb*RND could tolerate sequence variation at the 3′-ends of its substrates.

### Sequence preference and minimal length requirement of *Tb*RND

The RNA cleavage activity of RND-Exo is much weaker than that of RND-WT, however, similar to RND-WT, RND-Exo also showed some preference for U-rich sequences in the *in vitro* cleavage assay (Supplementary Figure S3B). Besides R0-R2 UMPs, RND-Exo also interacts with UMPs at the R3 and R4 positions of the substrate (Figures 3A-C and 4A-B). Different from R0–R3 UMPs, the nucleobase of R4 UMP forms extensive interactions with *Tb*RND (Figure 3C). Base-specific interactions were also observed for the UMPs at substrate R5 and R6 positions. R5 UMP is recognized by the ZF2 motif, whereas R6 UMP is bound at the shallow groove of the ZF1 motif (Figure 2B).

**Figure 6.**
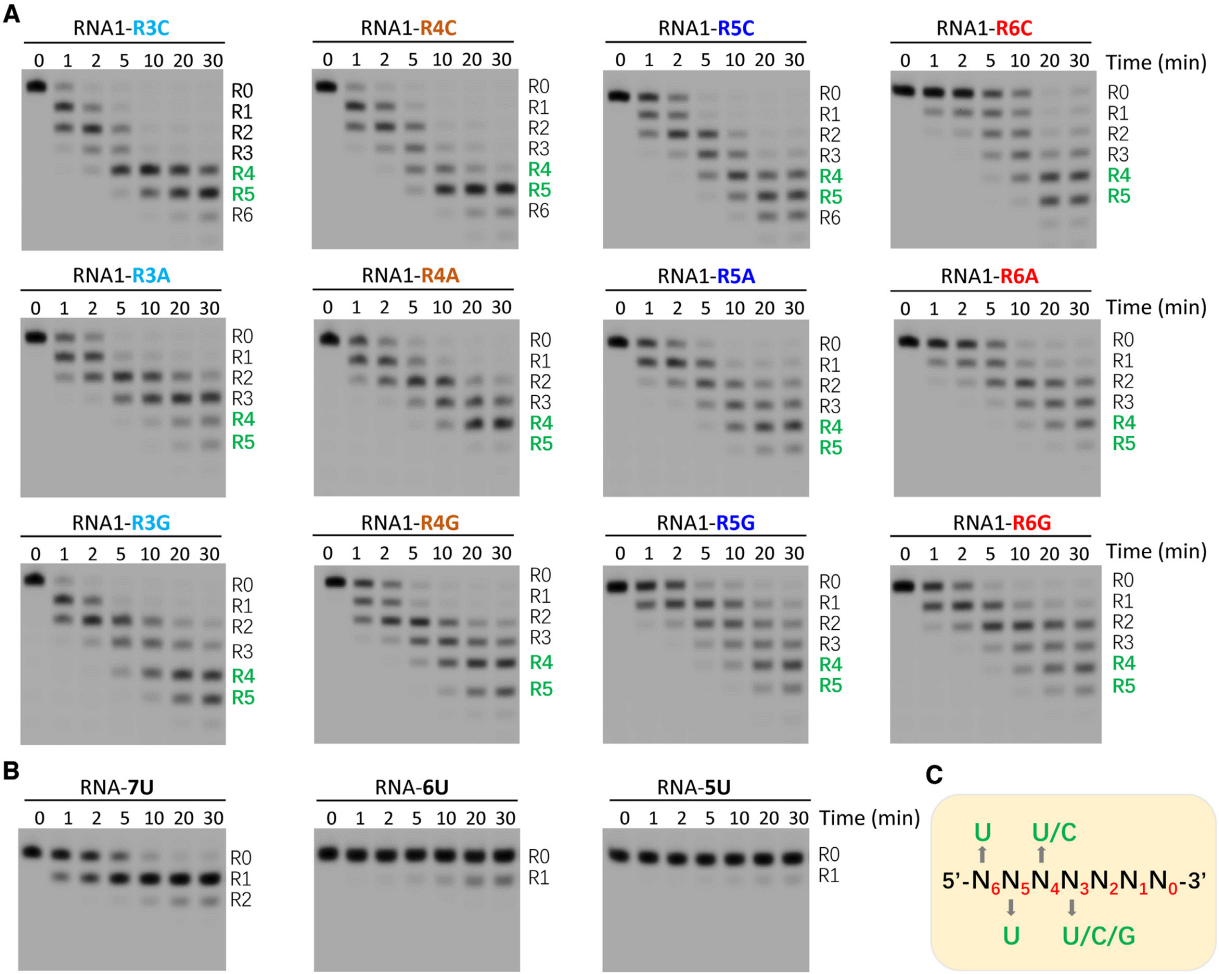
Impacts of sequence and length on RNA cleavage by *Tb*RND. (**A**) *In vitro* cleavage assays showing the impacts of R3-R6 nucleotides on RNA cleavage by *Tb*RND. (**B**) *In vitro* poly(U) RNA cleavage by *Tb*RND. (**C**) Schematic view showing the minimal substrate length requirement and nucleotide preference of *Tb*RND.

To investigate the impacts of individual nucleotides near the 3′ end of the substrate, we synthesized a series of RNAs. Except the mutations at the R3, R4, R5 or R6 positions, sequences of these RNAs are identical to that of RNA1 (Supplementary Table S4). As indicated by our ΔZF_3–4/RNA-12U complex structure and *in vitro* cleavage assays, upon the cleavage of R0 nucleotide, R1, R2 and other nucleotides will sequentially translocate to the active site and are cleaved by *Tb*RND. We performed *in vitro* cleavage assays using *Tb*RND and all RNA1 mutants (Figure 6A). Instead of 30-min reaction time, we mainly focused on the substrates or products at a reaction time of 1 min (Supplementary Table S6), which represents the initial stage of the reaction. R3 UMP does not form base-specific interaction with *Tb*RND (Figure 3A); in agree with this structural observation, substitution of R3 UMP by either CMP (for RNA1-R3C) or GMP (for RNA1-R3G) had no strong impact on RNA cleavage by *Tb*RND. However, as indicated by the higher percentage of intact substrate (37.17%), substitution of R3 UMP by AMP (for RNA1-R3A) inhibits the cleavage reaction. Substitution of R4 UMP by AMP (for RNA1-R4A) or GMP (for RNA1-R4G) also slowed down the reaction, but no clear difference was observed for CMP substitution (for RNA1-R4C). At a reaction time of 1 min, there had 45.81%, 44.98% and 13.80% intact substrates remained for RNA1-R4A, RNA1-R4G and RNA1-R4C, respectively. In contrast to R3 and R4 UMPs, substitution of R5 or R6-site UMPs by any other nucleotides all strongly inhibited the reaction. At a reaction time of 1 min, more than 49% intact substrates remained for all mutants. The strongest inhibition was observed for the RNA1-R6C mutant, which had 86.87% substrate remained uncleaved at a reaction time of 1 min.

In addition to RNA1 and RNA1 mutants, we also performed *in vitro* cleavage assays using three short poly(U) RNAs. As depicted in Figure 6B, *Tb*RND has very weak cleavage activities on RNA-5U (5′-UUUUU-3′) and RNA-6U (5′-UUUUUU-3′), whereas it can rapidly remove one uridine from the 3′ end of RNA-7U (5′-UUUUUUU-3′). In combination with the NMP-bound and ΔZF_3–4/RNA-12U complex structures, these *in vitro* cleavage assay results suggested that *Tb*RND has a minimal 7-nt substrate length requirement, and the apparent U-specificity of *Tb*RND is caused by the collective nucleotide requirement at the positions R3-R6 (Figure 6C), especially R6 that is bound at the shallow groove of ZF1. During *in vitro* RNA1 cleavage assays (Figure 1E), the major products had 4 or 5 uridines removed from the 3′ end. Although the length of the shorter product (5′-AACUUGUUU-3′, labelled as R5 on the gel) is longer than that of RNA-7U, it cannot be efficiently cleaved by *Tb*RND. Instead of the total length, structural and sequence analysis suggested that the 5′-end nucleotides (most likely the cytidine) prevent the product from further binding and cleavage by *Tb*RND.

### Structural comparison and implication of *Tb*RND

As indicated by the large cell parameter differences (Supplementary Table S3), the intermolecular packing of *Tb*RND is very different in the crystal lattices of the Apo-form and the RNA-complexed structures. Structural superposition showed that many RNA-interacting residues, including Arg179 of the Exo domain and Phe311 of ZF1, undergo large conformational changes to accommodate the bound RNA strand (Supplementary Figure S7A). In the presence of RNA-12U, Arg179 and Phe311 pack against the RNA and adopt a stable conformation, while in the absence of RNA, the side chains of Arg179 and Phe311 are disordered and flexible, as indicated by their high B-factors and weak electron densities.

Compared to the linker connecting ZF1 with ZF2 (amino acids 312–318), the Exo-ZF1 linker (amino acids 268–295) is much longer. Interestingly, however, the relative orientations between the Exo domain and ZF1 are very similar in the Apo-form and RNA-complexed structures of *Tb*RND (Supplementary Figure S7A). Structural comparison suggested that Arg175 and Ser176 play an important role in maintaining the relative orientation of Exo and ZF1 (Supplementary Figure S7B). Arg175 forms an H-bond interaction with the Gly304 of ZF1 via their main chain O and N atoms, respectively, while the side chain of Ser176 forms an H-bond interaction with Gly295, the last residue of the Exo-ZF1 linker. The relatively fixed orientations of the Exo domain and ZF1 were further elucidated in the G-form structure (Supplementary Figure S7C). In contrast to Exo and ZF1, the orientation of ZF2 is very flexible. ZF2 adopts an extended conformation in the Apo-form and the G-form structures (Supplementary Figure S2). In the RNA-complexed structure, ZF2 folds back and forms extensive interactions with the RNA at the 5′ end, which may help to kink and insert R6 nucleotide into the shallow groove of ZF1 (Supplementary Figure S7C).

*Ec*RND and *Sc*Rrp6 are the two founding members of the RND superfamily. *Ec*RND acts on tRNA, 5S rRNA, and some small-structured RNAs. *Sc*Rrp6 is a key component of the nuclear eukaryotic exosome. Like *Ec*RND, *Sc*Rrp6 also participates in processing and degradation of various RNAs, including tRNA, rRNA, and transcripts produced by RNA polymerase II. A previous study showed that the extra nucleotides following the 3′-end CCA will be cleaved during tRNA maturation and that variation of the extra nucleotides does not affect tRNA processing by *Ec*RND (66). *Ec*RND and *Sc*Rrp6 share a conserved central Exo domain with *Tb*RND, the sequence similarities between *Tb*RND Exo and those of *Ec*RND and *Sc*Rrp6 are about 40% (Supplementary Figure S8A). Superposition of the *Ec*RND and the ΔZF_3–4/RNA-12U complex structures showed that the overall fold of *Ec*RND Exo and *Tb*RND Exo are very similar (Supplementary Figure S8B). Like *Tb*RND, the active site pocket of *Ec*RND is wide open, which may contribute to the nucleotide tolerance of *Ec*RND.

**Figure 7.**
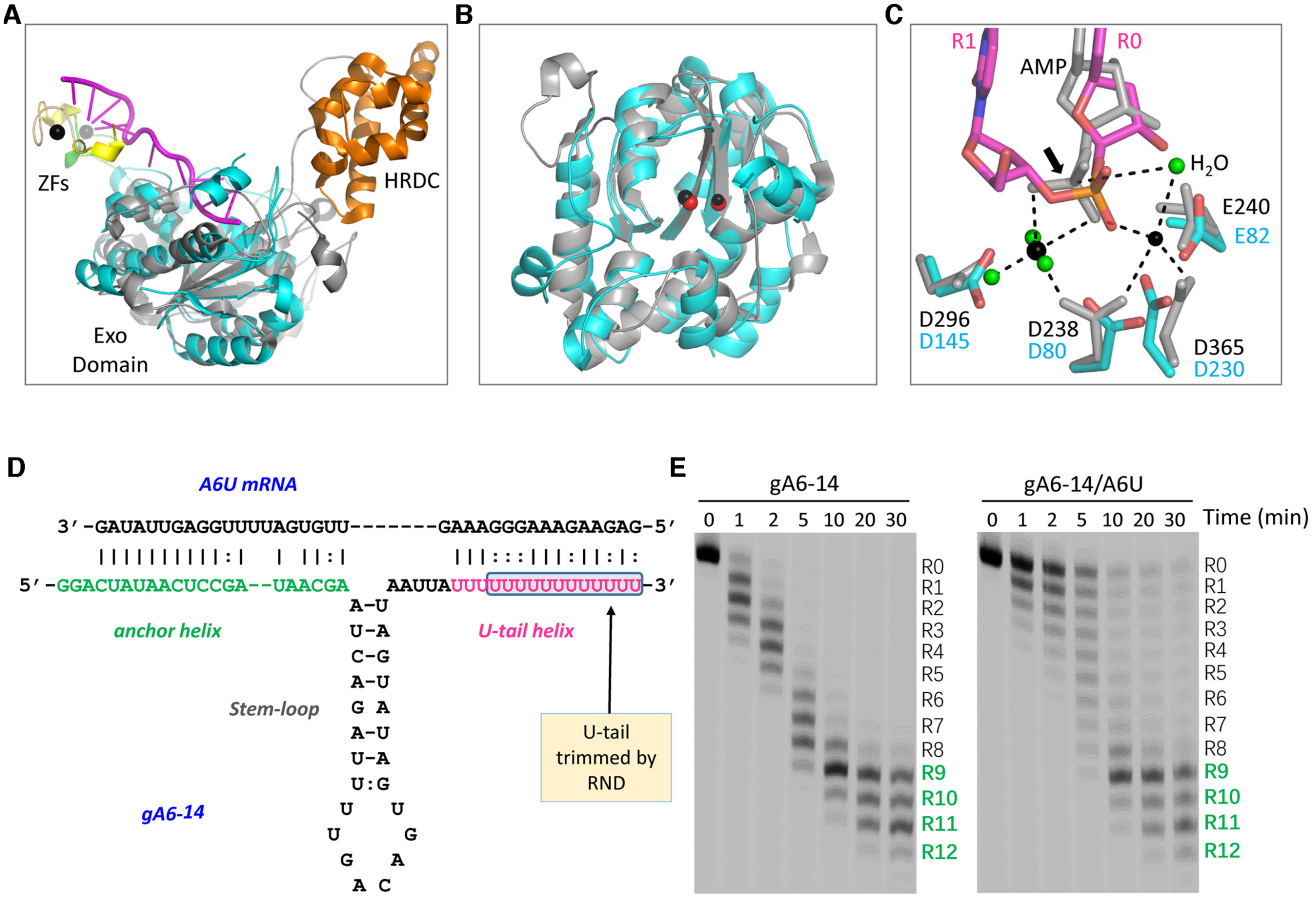
Structural comparison and *in vitro* gRNA cleavage by *Tb*RND. (**A**) Superposition of *Tb*RND ΔZF_3–4/RNA-12U with *Sc*Rrp6-AMP complexes. The ΔZF_3–4/RNA-12U complex is colored as in Figure 2A. The *Sc*Rrp6-AMP complex is colored in grey and orange for the Exo and HRDC domains, respectively. (**B**) Comparison of the Exo domains and the cations bound at the active sites. The cations are shown as red and black spheres for the ΔZF_3–4/RNA-12U and *Sc*Rrp6-AMP complexes, respectively. (**C**) Superposition of the catalytic site structures. For the *Sc*Rrp6-AMP complex, *Sc*Rrp6 and AMP are shown as grey sticks. Cations and the surrounding water molecules are shown as spheres in black and green, respectively. For the *Tb*RND ΔZF_3–4/RNA-12U complex, the C-atoms are colored in cyan and magenta for *Tb*RND and RNA, respectively. (**D**) Sequences and base pairing between gA16–14 gRNA and A6U mRNA. The U-tail trimmed by *Tb*RND is highlighted by a blue box. (**E**) *In vitro* gA6–14 gRNA cleavage by *Tb*RND.

To date, no substrate or product complex structure of *Ec*RND is available, however, AMP-bound and RNA-bound structures of *Sc*Rrp6 have been reported (57,67). Similar to *Ec*RND, *Sc*Rrp6 also shares a conserved fold for its Exo domain with *Tb*RND (Figure 7A). In the RNA-bound *Sc*Rrp6 structure, interaction between the R0- and R1-site nucleotides in the active site pocket are similar to that of *Tb*RND. However, possibly due to the relatively low resolution and use of an inactive *Sc*Rrp6 D238N mutant, only one cation was captured in the active site. Two cations were observed in the AMP-bound *Sc*Rrp6 structure. The orientation of the cations in the Rrp6-AMP and the ΔZF_3–4/RNA-12U complex structures are very similar (Figure 7B). AMP in the Rrp6-AMP structure adopts a similar conformation and mimics the R0 nucleotide in its interaction with the cations (Figure 6C). Taken together, these observations further confirm that the two-cation catalytic mechanism is shared by RND family members for RNA cleavage.

*Ec*RND and *Sc*Rrp6 possess an HRDC domain at their C-termini. Similar to *Tb*RND ZF2, the HRDC domains of *Ec*RND and *Sc*Rrp6 can undergo large conformational changes (Supplementary Figure S8C). However, the lack of sequence and structural similarity with HRDC and the fixed orientation of ZF1 all indicated that *Tb*RND is unique among RND family proteins. Previous study showed that *Tb*RND specifically acts on the 3′ U-tails of gRNAs *in vivo* (55). In addition to U-tails, gRNAs also contain an anchor domain and a guide domain. Via base-pairing, gRNA and its cognate mRNA will form a structure composed of two helixes and one stem-loop. To investigate whether these structural features and pairing with cognate mRNAs will affect gRNA cleavage by *Tb*RND, we synthesized gA6–14 gRNA and the cognate A6U mRNA (Figure 7D), which are widely used in the mRNA editing studies (32). As showed by the *in vitro* cleavage assays (Figure 7E), *Tb*RND can efficiently remove uridines from the 3′ end of gA6–14 in the absence of A6U. Introducing of A6U slightly slowed down the reaction. However, may be due to the dynamic pairing between gA6–14 U-tail and the purine-rich region of A6U, A6U could not fully prevent gA6–14 from cleavage by *Tb*RND. Independent of the presence or absence of A6U mRNA, the major gA6–14 cleavage products had 9–11 uridines removed from the 3′ end at a reaction time of 30 min.

## DISCUSSION

RND superfamily proteins play important roles in RNA processing and degradation, of which *Tb*RND is a unique member. *Tb*RND localizes in the mitochondria of *T. brucei* and is the first reported organellar RND (55). Previous study suggested that *Tb*RND is likely involved in the metabolism of gRNA. Silencing of *Tb*RND leads to extended gRNA U-tails, whereas, overexpression of *Tb*RND results in total gRNA depletion and inhibition of RNA editing. Here, we report the structural and functional studies of *Tb*RND. Several structures were determined, including a ΔZF_3–4/RNA-12U complex (Figure 2A), which represents the first ternary structure composed of native RND protein, substrate RNA, and coordinating cations. The RNA cleavage activity of *Tb*RND is divalent cation-dependent, as shown by the coordination of two such cations by conserved catalytic residues in the Exo domain of the complex structure. In addition to *Tb*RND, structural comparison and analysis further confirmed that RND members all use a similar two-cation-assisted mechanism for catalysis (Figure 4D). Indeed, two-cation catalytic mechanisms are also observed for many other nucleases, including RNase H (63) and RNase III (64), indicating that it is a very common nucleic acid cleavage mechanism.

Our *in vitro* assays showed that the RNA cleavage activity of *Tb*RND occurs in the Exo domain (Supplementary Figure S3B), which is conserved and adopts a similar fold to the RND superfamily protein structures, including *Ec*RND (56) and *Sc*Rrp6 (57). Although it needs to be further verified, the structural similarity suggested that the RND Exo domain has no strong nucleotide preference at the active site. This may explain why *Ec*RND and *Sc*Rrp6 can function on various types of RNAs, including tRNA, rRNA, and other structured RNAs, all of which possess different sequences at their 3′-ends (66).

*Tb*RND possesses a ZFD domain at its C-terminus, which distinguishes it from all other RND members. Interestingly, besides *Tb*RND, *T. brucei* expresses an additional RND type protein, *Tb*Rrp6 (55). *Tb*Rrp6 is both nuclear and cytosolic, and unlike *Tb*RND, is more similar to *Sc*Rrp6 in domain architecture, as it contains a HRDC domain at the C-terminus. The HRDC domain is also present in many other nucleic acid binding and processing proteins, such as helicase RecQ (68,69). The flexibility of the HRDC domain may afford related proteins the capability for binding different types of nucleic acids. Similar to HRDC, ZFDs are also common nucleic acid binding domains. However, *Tb*RND ZFD utilizes a unique substrate binding mode (Figure 2B), in which the shallow grooves of *Tb*RND ZF1–4 directly participate in RNA binding and uridine selection (Figures 2B and 5A–C).

Interestingly, although the HRDC domain of *Sc*Rrp6 can undergo large conformational changes, it forms stable interactions with the Exo domain, independent of the presence or absence of AMP (67). Disruption of the Exo-HRDC interaction will affect the processing of RNAs that need to be precisely trimmed, such as snR40. As revealed by our structural comparison, the ZF2 motif of *Tb*RND structure is flexible, but the ZF1 motif and the Exo domain adopt a relatively fixed orientation (Supplementary Figure S7). Like the Exo-HRDC interaction of *Sc*Rrp6, the Exo-ZF1 interaction of *Tb*RND might have evolved to facilitate the binding and cleavage of gRNAs.

Besides gRNAs, other *T. brucei* RNAs, such as 9 S and 12 S rRNA, are also uridylated at their 3′-ends (45,70). However, an *in vivo* study indicated that *Tb*RND specifically works on gRNAs (55). While our structures suggested that *Tb*RND has preference for U-rich RNAs (Figures 5A–C), it is very unlikely that *Tb*RND can discriminate other uridylated RNAs from gRNAs on its own. In eukaryotes, RND members, such as *Sc*Rrp6, can associate with the core exosome to ensure a higher level of regulation. It is well-known that some members of the mitochondrial RNA binding complex 1 (MRB1), such as GAP1 and GAP2, can bind and stabilize gRNAs (71,72). In the future, it is worth investigating whether these gRNA-binding proteins can interact with *Tb*RND and affect its substrate specificity. gRNA U-tails are important for interacting and editing of the cognate mRNAs (32,36). However, as supported by our *in vitro* cleavage assay, neither the anchor and guide domains of gRNAs nor pairing with their cognate mRNAs could prevent gRNA U-tails from cleavage by *Tb*RND (Figure 7D-E). We speculated that *Tb*RND may only function at the gRNA degradation state; *Tb*RND removes the uridines from the 3′ ends of gRNAs, the resulting products will be further degraded by other nucleases. During the mRNA editing state, interacting with the editing complexes prevent gRNA from cleavage by *Tb*RND. When overexpressed, *Tb*RND may be able to complete with the editing complexes, triggering gRNA degradation and subsequent inhibition of mRNA editing.

In conclusion, we report a structural and functional study of *Tb*RND, describing a high-resolution structure composed of native *Tb*RND, substrate RNA, and coordinating cations. In addition to confirming the two-metal catalytic mechanism shared by all RND members, our structures also revealed the detailed basis for gRNA U-tail binding by *Tb*RND ZFD. The ZFD is absent in *Ec*RND and *Sc*Rrp6, but ZFD-containing RNDs are common in many trypanosomatids, from the most basal-branch containing *Paratrypanosoma confusum* to the deadly human disease causative agents, including *T. cruzi* and *Leishmania donovani*. The sequence similarities (Supplementary Figure S9) between *Tb*RND and these trypanosoma RND proteins are very high (>80%), suggesting that these RNDs may adopt similar folds and possess similar RNA cleavage activity. In the future, it is worth further investigating the functions played by RND proteins in *T. brucei* and the related Trypanosoma.

## DATA AVAILABILITY

Structural factors and coordinates have been deposited in the Protein Data Bank under accession codes 7C42, 7C4C, 7C43, 7C47, 7C4B and 7C45 for the Apo-form, GMP-bound, AMP-bound, CMP-bound, UMP-bound and RNA-complexed *Tb*RND structures, respectively.

## Supplementary Material

gkaa1197_Supplemental_File

## References

[1] Stuart K., Brun R., Croft S., Fairlamb A., Gurtler R.E., McKerrow J., Reed S., Tarleton R. Kinetoplastids: related protozoan pathogens, different diseases. J. Clin. Invest. 2008; 118:1301–1310. 18382742 10.1172/JCI33945 PMC2276762

[2] Kagbadouno M.S., Camara M., Rouamba J., Rayaisse J.B., Traore I.S., Camara O., Onikoyamou M.F., Courtin F., Ravel S., de Meeus T. et al. Epidemiology of sleeping sickness in Boffa (Guinea): where are the trypanosomes. PLoS Negl. Trop. Dis. 2012; 6:e1949. 23272259 10.1371/journal.pntd.0001949 PMC3521671

[3] Lundkvist G.B., Kristensson K., Bentivoglio M. Why trypanosomes cause sleeping sickness. Physiology (Bethesda). 2004; 19:198–206. 15304634 10.1152/physiol.00006.2004

[4] Vickerman K. Polymorphism and mitochondrial activity in sleeping sickness trypanosomes. Nature. 1965; 208:762–766. 5868887 10.1038/208762a0

[5] Welburn S.C., Picozzi K., Fevre E.M., Coleman P.G., Odiit M., Carrington M., Maudlin I. Identification of human-infective trypanosomes in animal reservoir of sleeping sickness in Uganda by means of serum-resistance-associated (SRA) gene. Lancet. 2001; 358:2017–2019. 11755607 10.1016/s0140-6736(01)07096-9

[6] Meyers A.C., Purnell J.C., Ellis M.M., Auckland L.D., Meinders M., Hamer S.A. Nationwide exposure of U.S. working dogs to the chagas disease parasite, Trypanosoma cruzi. Am. J. Trop. Med. Hyg. 2020; 102:1078–1085. 32189615 10.4269/ajtmh.19-0582 PMC7204581

[7] El-Sayed N.M., Myler P.J., Bartholomeu D.C., Nilsson D., Aggarwal G., Tran A.N., Ghedin E., Worthey E.A., Delcher A.L., Blandin G. et al. The genome sequence of Trypanosoma cruzi, etiologic agent of Chagas disease. Science. 2005; 309:409–415. 16020725 10.1126/science.1112631

[8] Miles M.A., Souza A., Povoa M., Shaw J.J., Lainson R., Toye P.J. Isozymic heterogeneity of Trypanosoma cruzi in the first autochthonous patients with Chagas' disease in Amazonian Brazil. Nature. 1978; 272:819–821. 417267 10.1038/272819a0

[9] Medley G.F., Hollingsworth T.D., Olliaro P.L., Adams E.R. Health-seeking behaviour, diagnostics and transmission dynamics in the control of visceral leishmaniasis in the Indian subcontinent. Nature. 2015; 528:S102–S108. 26633763 10.1038/nature16042

[10] de Gorgolas M., Miles M.A. Visceral leishmaniasis and AIDS. Nature. 1994; 372:734. 10.1038/372734b0 7997260

[11] Lainson R., Shaw J.J. Epidemiology and ecology of leishmaniasis in Latin-America. Nature. 1978; 273:595–600. 351409 10.1038/273595a0

[12] Steverding D. The history of African trypanosomiasis. Parasit Vectors. 2008; 1:3. 18275594 10.1186/1756-3305-1-3 PMC2270819

[13] Molina J.P., Madi R.R., Solferini V.N., Ceccarelli P.S., Pinheiro H.P., Ueta M.T. Trypanosomatids (Protozoa: Kinetoplastida) in three species of Armored Catfish from Mogi-Guacu river, Pirassununga, Sao Paulo, Brazil. Rev Bras Parasitol Vet. 2016; 25:131–141. 27334813 10.1590/S1984-29612016027

[14] Lukes J., Butenko A., Hashimi H., Maslov D.A., Votypka J., Yurchenko V. Trypanosomatids are much more than just Trypanosomes: clues from the expanded family tree. Trends Parasitol. 2018; 34:466–480. 29605546 10.1016/j.pt.2018.03.002

[15] Shapiro T.A., Englund P.T. The structure and replication of kinetoplast DNA. Annu. Rev. Microbiol. 1995; 49:117–143. 8561456 10.1146/annurev.mi.49.100195.001001

[16] Chen J., Rauch C.A., White J.H., Englund P.T., Cozzarelli N.R. The topology of the kinetoplast DNA network. Cell. 1995; 80:61–69. 7813018 10.1016/0092-8674(95)90451-4

[17] Chen J., Englund P.T., Cozzarelli N.R. Changes in network topology during the replication of kinetoplast DNA. EMBO J. 1995; 14:6339–6347. 8557054 10.1002/j.1460-2075.1995.tb00325.x PMC394759

[18] Chen K.K., Donelson J.E. Sequences of two kinetoplast DNA minicircles of Tryptanosoma brucei. Proc. Natl. Acad. Sci. USA. 1980; 77:2445–2449. 6930643 10.1073/pnas.77.5.2445 PMC349416

[19] Clayton D.A. Replication and transcription of vertebrate mitochondrial DNA. Annu. Rev. Cell Biol. 1991; 7:453–478. 1809353 10.1146/annurev.cb.07.110191.002321

[20] Carpenter L.R., Englund P.T. Kinetoplast maxicircle DNA replication in Crithidia fasciculata and Trypanosoma brucei. Mol. Cell. Biol. 1995; 15:6794–6803. 8524245 10.1128/mcb.15.12.6794 PMC230933

[21] Feagin J.E., Jasmer D.P., Stuart K. Apocytochrome b and other mitochondrial DNA sequences are differentially expressed during the life cycle of Trypanosoma brucei. Nucleic Acids Res. 1985; 13:4577–4596. 2409537 10.1093/nar/13.12.4577 PMC321807

[22] Grams J., McManus M.T., Hajduk S.L. Processing of polycistronic guide RNAs is associated with RNA editing complexes in Trypanosoma brucei. EMBO J. 2000; 19:5525–5532. 11032819 10.1093/emboj/19.20.5525 PMC314002

[23] Koslowsky D.J., Yahampath G. Mitochondrial mRNA 3′ cleavage/polyadenylation and RNA editing in Trypanosoma brucei are independent events. Mol. Biochem. Parasitol. 1997; 90:81–94. 9497034 10.1016/s0166-6851(97)00133-3

[24] Aphasizheva I., Alfonzo J., Carnes J., Cestari I., Cruz-Reyes J., Goringer H.U., Hajduk S., Lukes J., Madison-Antenucci S., Maslov D.A. et al. Lexis and grammar of mitochondrial RNA processing in Trypanosomes. Trends Parasitol. 2020; 36:337–355. 32191849 10.1016/j.pt.2020.01.006 PMC7083771

[25] Read L.K., Lukes J., Hashimi H. Trypanosome RNA editing: the complexity of getting U in and taking U out. Wiley Interdiscip. Rev. RNA. 2016; 7:33–51. 26522170 10.1002/wrna.1313 PMC4835692

[26] Cruz-Reyes J., Mooers B.H.M., Doharey P.K., Meehan J., Gulati S. Dynamic RNA holo-editosomes with subcomplex variants: Insights into the control of trypanosome editing. Wires RNA. 2018; 9:e1502. 30101566 10.1002/wrna.1502 PMC6185801

[27] Byrne E.M., Connell G.J., Simpson L. Guide RNA-directed uridine insertion RNA editing in vitro. EMBO J. 1996; 15:6758–6765. 8978701 PMC452499

[28] Hermann T., Schmid B., Heumann H., Goringer H.U. A three-dimensional working model for a guide RNA from Trypanosoma brucei. Nucleic Acids Res. 1997; 25:2311–2318. 9171080 10.1093/nar/25.12.2311 PMC146733

[29] Kable M.L., Seiwert S.D., Heidmann S., Stuart K. RNA editing: a mechanism for gRNA-specified uridylate insertion into precursor mRNA. Science. 1996; 273:1189–1195. 8703045 10.1126/science.273.5279.1189

[30] Maslov D.A., Simpson L. The polarity of editing within a multiple gRNA-mediated domain is due to formation of anchors for upstream gRNAs by downstream editing. Cell. 1992; 70:459–467. 1379519 10.1016/0092-8674(92)90170-h

[31] Cruz-Reyes J., Zhelonkina A., Rusche L., Sollner-Webb B. Trypanosome RNA editing: Simple guide RNA features enhance U deletion 100-fold. Mol. Cell. Biol. 2001; 21:884–892. 11154275 10.1128/MCB.21.3.884-892.2001 PMC86679

[32] Koslowsky D.J., Reifur L., Yu L.E., Chen W. Evidence for U-tail stabilization of gRNA/mRNA interactions in kinetoplastid RNA editing. RNA Biol. 2004; 1:28–34. 17194935 10.4161/rna.1.1.898 PMC2762388

[33] Leung S.S., Koslowsky D.J. RNA editing in Trypanosoma brucei: characterization of gRNA U-tail interactions with partially edited mRNA substrates. Nucleic Acids Res. 2001; 29:703–709. 11160892 10.1093/nar/29.3.703 PMC30404

[34] Leung S.S., Koslowsky D.J. Mapping contacts between gRNA and mRNA in trypanosome RNA editing. Nucleic Acids Res. 1999; 27:778–787. 9889273 10.1093/nar/27.3.778 PMC148247

[35] Mooers B.H., Singh A. The crystal structure of an oligo(U):pre-mRNA duplex from a trypanosome RNA editing substrate. RNA. 2011; 17:1870–1883. 21878548 10.1261/rna.2880311 PMC3185919

[36] Seiwert S.D., Heidmann S., Stuart K. Direct visualization of uridylate deletion in vitro suggests a mechanism for kinetoplastid RNA editing. Cell. 1996; 84:831–841. 8601307 10.1016/s0092-8674(00)81062-4

[37] Kelly F.D., Sanchez M.A., Landfear S.M. Touching the surface: diverse roles for the flagellar membrane in kinetoplastid parasites. Microbiol. Mol. Biol. Rev. 2020; 84:e00079-19. 32238446 10.1128/MMBR.00079-19 PMC7117551

[38] Landfear S.M., Zilberstein D. Sensing what's out there - kinetoplastid parasites. Trends Parasitol. 2019; 35:274–277. 30655057 10.1016/j.pt.2018.12.004 PMC7392150

[39] De Pablos L.M. Editorial: New insights into the genomes of kinetoplastid parasites. Curr. Genomics. 2018; 19:77. 29491735 10.2174/138920291902180112113521 PMC5814964

[40] Tiengwe C., Marques C.A., McCulloch R. Nuclear DNA replication initiation in kinetoplastid parasites: new insights into an ancient process. Trends Parasitol. 2014; 30:27–36. 24287149 10.1016/j.pt.2013.10.009

[41] Price H.P., MacLean L., Marrison J., O’Toole P.J., Smith D.F. Validation of a new method for immobilising kinetoplastid parasites for live cell imaging. Mol. Biochem. Parasitol. 2010; 169:66–69. 19815033 10.1016/j.molbiopara.2009.09.008 PMC2791879

[42] Osato D., Rogers K., Guo Q., Li F., Richmond G., Klug F., Simpson L. Uridine insertion/deletion RNA editing in trypanosomatid mitochondria: In search of the editosome. RNA. 2009; 15:1338–1344. 19447916 10.1261/rna.1642809 PMC2704074

[43] Gao G., Rogers K., Li F., Guo Q., Osato D., Zhou S.X., Falick A.M., Simpson L. Uridine insertion/deletion RNA editing in Trypanosomatids: specific stimulation in vitro of Leishmania tarentolae REL1 RNA ligase activity by the MP63 zinc finger protein. Protist. 2010; 161:489–496. 20138580 10.1016/j.protis.2010.01.001 PMC2864329

[44] Aphasizheva I., Ringpis G.E., Weng J., Gershon P.D., Lathrop R.H., Aphasizhev R. Novel TUTase associates with an editosome-like complex in mitochondria of Trypanosoma brucei. RNA. 2009; 15:1322–1337. 19465686 10.1261/rna.1538809 PMC2704088

[45] Aphasizheva I., Aphasizhev R. RET1-catalyzed uridylylation shapes the mitochondrial transcriptome in Trypanosoma brucei. Mol. Cell. Biol. 2010; 30:1555–1567. 20086102 10.1128/MCB.01281-09 PMC2832499

[46] Suematsu T., Zhang L., Aphasizheva I., Monti S., Huang L., Wang Q., Costello C.E., Aphasizhev R. Antisense transcripts delimit exonucleolytic activity of the mitochondrial 3′ processome to generate guide RNAs. Mol. Cell. 2016; 61:364–378. 26833087 10.1016/j.molcel.2016.01.004 PMC4744118

[47] Ibrahim F., Rymarquis L.A., Kim E.J., Becker J., Balassa E., Green P.J., Cerutti H. Uridylation of mature miRNAs and siRNAs by the MUT68 nucleotidyltransferase promotes their degradation in Chlamydomonas. Proc. Natl. Acad. Sci. U.S.A. 2010; 107:3906–3911. 20142471 10.1073/pnas.0912632107 PMC2840426

[48] Ramachandran V., Chen X. Degradation of microRNAs by a family of exoribonucleases in Arabidopsis. Science. 2008; 321:1490–1492. 18787168 10.1126/science.1163728 PMC2570778

[49] Rissland O.S., Norbury C.J. Decapping is preceded by 3′ uridylation in a novel pathway of bulk mRNA turnover. Nat. Struct. Mol. Biol. 2009; 16:616–623. 19430462 10.1038/nsmb.1601 PMC2875167

[50] Wickens M., Kwak J.E. Molecular biology. A tail tale for U. Science. 2008; 319:1344–1345. 18323438 10.1126/science.1154946

[51] Riley G.R., Corell R.A., Stuart K. Multiple guide RNAs for identical editing of Trypanosoma brucei apocytochrome b mRNA have an unusual minicircle location and are developmentally regulated. J. Biol. Chem. 1994; 269:6101–6108. 7509798

[52] Rogers K., Gao G., Simpson L. Uridylate-specific 3′ 5′-exoribonucleases involved in uridylate-deletion RNA editing in trypanosomatid mitochondria. J. Biol. Chem. 2007; 282:29073–29080. 17699520 10.1074/jbc.M704551200

[53] Kang X., Rogers K., Gao G., Falick A.M., Zhou S., Simpson L. Reconstitution of uridine-deletion precleaved RNA editing with two recombinant enzymes. Proc. Natl. Acad. Sci. U.S.A. 2005; 102:1017–1022. 15657144 10.1073/pnas.0409275102 PMC545852

[54] Niemann M., Kaibel H., Schluter E., Weitzel K., Brecht M., Goringer H.U. Kinetoplastid RNA editing involves a 3′ nucleotidyl phosphatase activity. Nucleic Acids Res. 2009; 37:1897–1906. 19190092 10.1093/nar/gkp049 PMC2665232

[55] Zimmer S.L., McEvoy S.M., Li J., Qu J., Read L.K. A novel member of the RNase D exoribonuclease family functions in mitochondrial guide RNA metabolism in Trypanosoma brucei. J. Biol. Chem. 2011; 286:10329–10340. 21252235 10.1074/jbc.M110.152439 PMC3060487

[56] Zuo Y., Wang Y., Malhotra A. Crystal structure of Escherichia coli RNase D, an exoribonuclease involved in structured RNA processing. Structure. 2005; 13:973–984. 16004870 10.1016/j.str.2005.04.015

[57] Wasmuth E.V., Januszyk K., Lima C.D. Structure of an Rrp6-RNA exosome complex bound to poly(A) RNA. Nature. 2014; 511:435–439. 25043052 10.1038/nature13406 PMC4310248

[58] Minor W., Cymborowski M., Otwinowski Z., Chruszcz M. HKL-3000: the integration of data reduction and structure solution–from diffraction images to an initial model in minutes. Acta Crystallogr. D, Biol. Crystallogr. 2006; 62:859–866. 16855301 10.1107/S0907444906019949

[59] Giacovazzo C., Siliqi D. Phasing via SAD/MAD data: the method of the joint probability distribution functions. Acta Crystallogr. D, Biol. Crystallogr. 2004; 60:73–82. 14684895 10.1107/s0907444903022406

[60] Adams P.D., Grosse-Kunstleve R.W., Hung L.W., Ioerger T.R., McCoy A.J., Moriarty N.W., Read R.J., Sacchettini J.C., Sauter N.K., Terwilliger T.C. PHENIX: building new software for automated crystallographic structure determination. Acta Crystallogr. D, Biol. Crystallogr. 2002; 58:1948–1954. 12393927 10.1107/s0907444902016657

[61] Potterton E., Briggs P., Turkenburg M., Dodson E. A graphical user interface to the CCP4 program suite. Acta Crystallogr. D, Biol. Crystallogr. 2003; 59:1131–1137. 12832755 10.1107/s0907444903008126

[62] Emsley P., Cowtan K. Coot: model-building tools for molecular graphics. Acta Crystallogr. D, Biol. Crystallogr. 2004; 60:2126–2132. 15572765 10.1107/S0907444904019158

[63] Nowotny M., Gaidamakov S.A., Crouch R.J., Yang W. Crystal structures of RNase H bound to an RNA/DNA hybrid: substrate specificity and metal-dependent catalysis. Cell. 2005; 121:1005–1016. 15989951 10.1016/j.cell.2005.04.024

[64] Gan J., Tropea J.E., Austin B.P., Court D.L., Waugh D.S., Ji X. Structural insight into the mechanism of double-stranded RNA processing by ribonuclease III. Cell. 2006; 124:355–366. 16439209 10.1016/j.cell.2005.11.034

[65] Zhang J., Liu H., Yao Q., Yu X., Chen Y., Cui R., Wu B., Zheng L., Zuo J., Huang Z. et al. Structural basis for single-stranded RNA recognition and cleavage by C3PO. Nucleic Acids Res. 2016; 44:9494–9504. 27596600 10.1093/nar/gkw776 PMC5100593

[66] Cudny H., Zaniewski R., Deutscher M.P. Escherichia coli RNase D. Catalytic properties and substrate specificity. J. Biol. Chem. 1981; 256:5633–5637. 6263886

[67] Midtgaard S.F., Assenholt J., Jonstrup A.T., Van L.B., Jensen T.H., Brodersen D.E. Structure of the nuclear exosome component Rrp6p reveals an interplay between the active site and the HRDC domain. Proc. Natl. Acad. Sci. U.S.A. 2006; 103:11898–11903. 16882719 10.1073/pnas.0604731103 PMC2131688

[68] Killoran M.P., Keck J.L. Structure and function of the regulatory C-terminal HRDC domain from Deinococcus radiodurans RecQ. Nucleic Acids Res. 2008; 36:3139–3149. 18411208 10.1093/nar/gkn143 PMC2396406

[69] Bernstein D.A., Keck J.L. Conferring substrate specificity to DNA helicases: role of the RecQ HRDC domain. Structure. 2005; 13:1173–1182. 16084389 10.1016/j.str.2005.04.018

[70] Adler B.K., Harris M.E., Bertrand K.I., Hajduk S.L. Modification of Trypanosoma brucei mitochondrial rRNA by posttranscriptional 3′ polyuridine tail formation. Mol. Cell. Biol. 1991; 11:5878–5884. 1719373 10.1128/mcb.11.12.5878-5884.1991 PMC361737

[71] Weng J., Aphasizheva I., Etheridge R.D., Huang L., Wang X., Falick A.M., Aphasizhev R. Guide RNA-binding complex from mitochondria of trypanosomatids. Mol. Cell. 2008; 32:198–209. 18951088 10.1016/j.molcel.2008.08.023 PMC2645705

[72] Hashimi H., Cicova Z., Novotna L., Wen Y.Z., Lukes J. Kinetoplastid guide RNA biogenesis is dependent on subunits of the mitochondrial RNA binding complex 1 and mitochondrial RNA polymerase. RNA. 2009; 15:588–599. 19228586 10.1261/rna.1411809 PMC2661843

