# Supplementary material for: Structural basis for guide RNA trimming by RNase D ribonuclease in Trypanosoma brucei
Source: Nucleic Acids Res. 2020 Dec 17;49(1):568–83. doi: 10.1093/nar/gkaa1197 (PMC7797062; doi:10.1093/nar/gkaa1197)
Supplement: gkaa1197_Supplemental_File [file gkaa1197_supplemental_file.pdf]

Structural basis for guide RNA trimming by RNase D ribonuclease in *Trypanosoma brucei*

Yanqing Gao[1], Hehua Liu[1,2],Chong Zhang[3], Shichen Su[2], Yiqing Chen[1], Xi Chen[1,2], Yangyang Li[1], Zhiwei Shao[1], Yixi Zhang[1], Qiyuan Shao[1], Jixi Li[1], Zhen Huang[3], Jinbiao Ma[2], Jianhua Gan[1,*]

[1]Shanghai Public Health Clinical Center, State Key Laboratory of Genetic Engineering, Collaborative Innovation Center of Genetics and Development, Department of Physiology and Biophysics, School of Life Sciences, Fudan University, Shanghai 200438, China.

[2]State Key Laboratory of Genetic Engineering, Collaborative Innovation Center of Genetics and Development, Department of Biochemistry, School of Life Sciences, Fudan University, Shanghai 200438, China.

[3]College of Life Sciences, Sichuan University, Chengdu 610041, China.

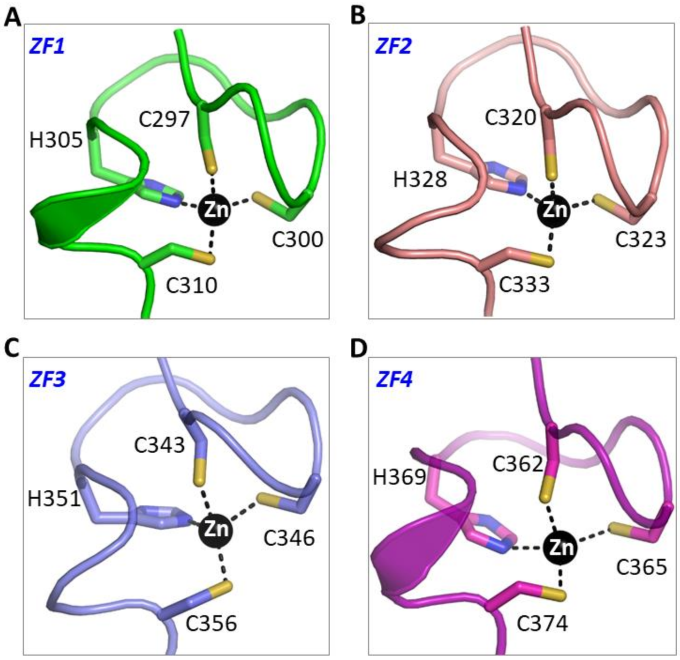

Fig. S1: Overall fold and Zinc-coordination of the *Tb*RND ZF motifs.

Cartoon depiction of ZF1, ZF2, ZF3, and ZF4. Zinc ions and Zinc-coordinating residues are shown as black spheres and sticks in atomic colors (N, blue; S, yellow).

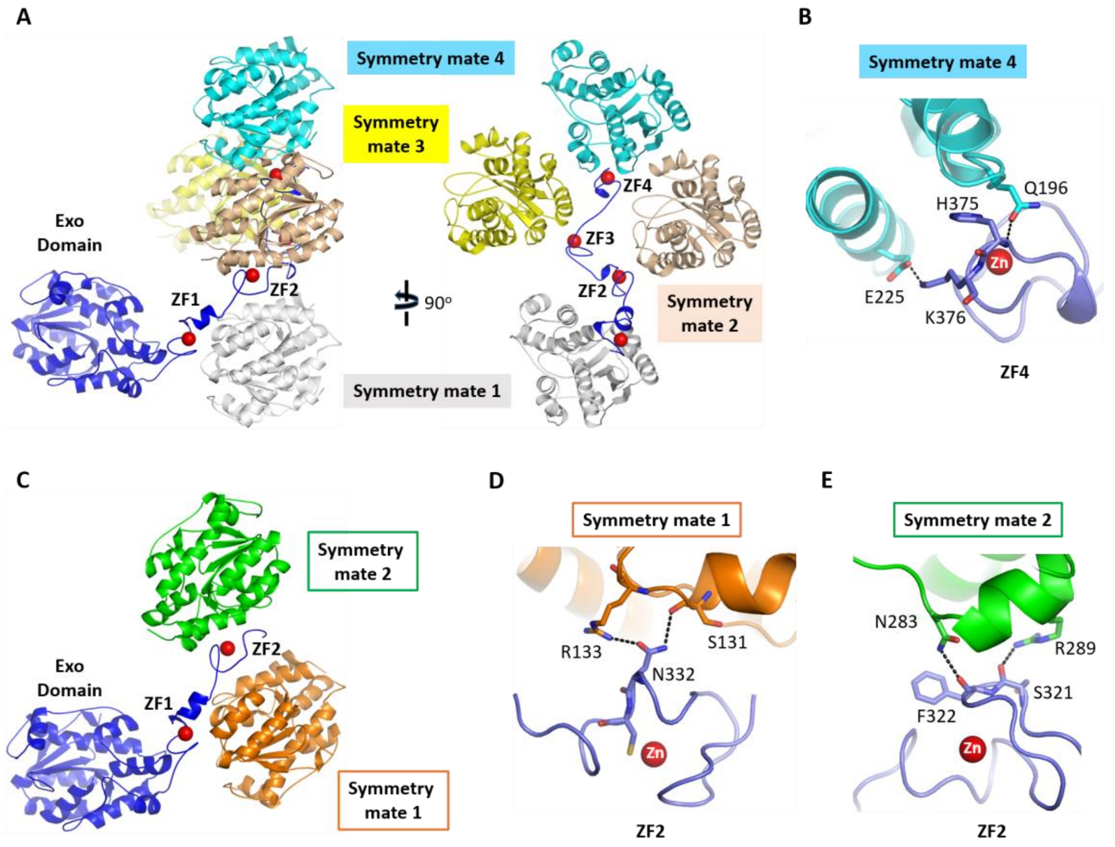

Fig. S2: Crystal packing and interactions that stabilized the extended ZFD conformations in the *Tb*RND structures.

(**A**) Packing between *Tb*RND and symmetry-related molecules in the G-form structure. (**B**) H-bond interactions between *Tb*RND ZF4 and the Exo domain of symmetry-related *Tb*RND in the G-form structure. (**C**) Packing between *Tb*RND and symmetry-related molecules in the Apo-form structure. (**D-E**) H-bond interactions between *Tb*RND ZF2 and the Exo domains of symmetry-related *Tb*RNDs in the Apo-form structure. *Tb*RND and Zn^{2+} ions are shown as blue cartoon and red spheres in both structure. The symmetry-related *Tb*RND molecules are shown as cartoon in white, wheat, yellow, and cyan in the G-form structure, whereas they are colored in orange and green in the Apo-form structure. For clarity, the ZFD domains of all symmetry-related *Tb*RND molecules are omitted.

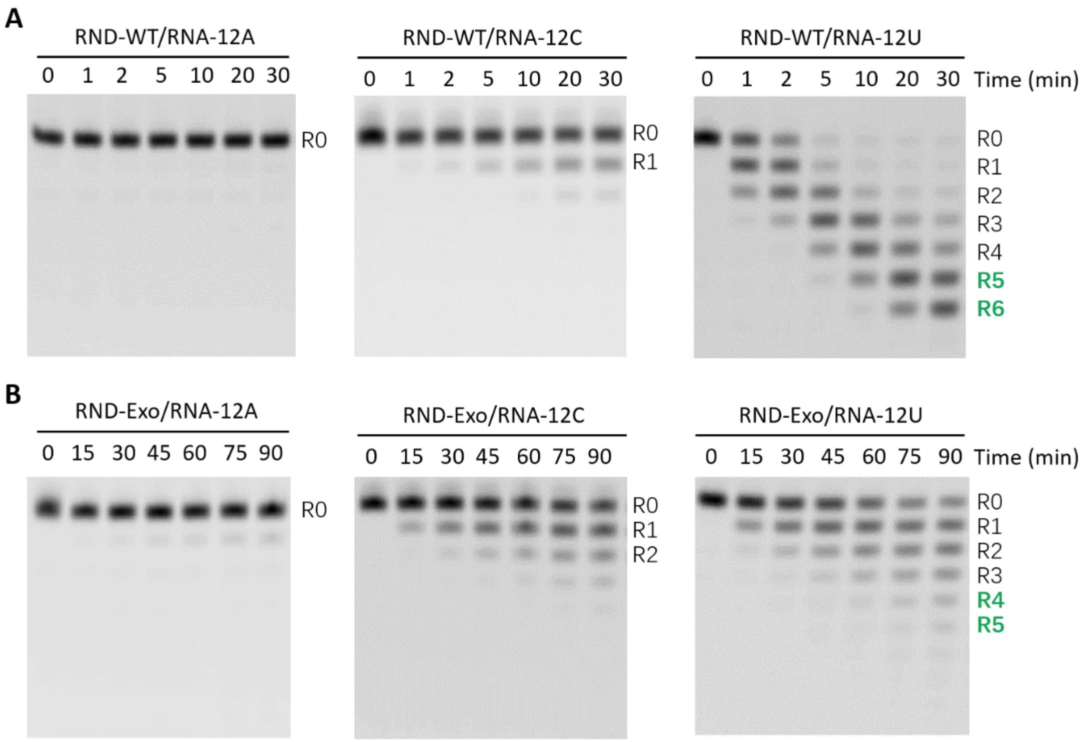

Fig. S3: Comparison of RNA cleavage catalyzed by *Tb*RND.

(**A**) *In vitro* RNA cleavage reaction catalyzed by RND-WT. (**B**) *In vitro* RNA cleavage reaction catalyzed by RND-Exo.

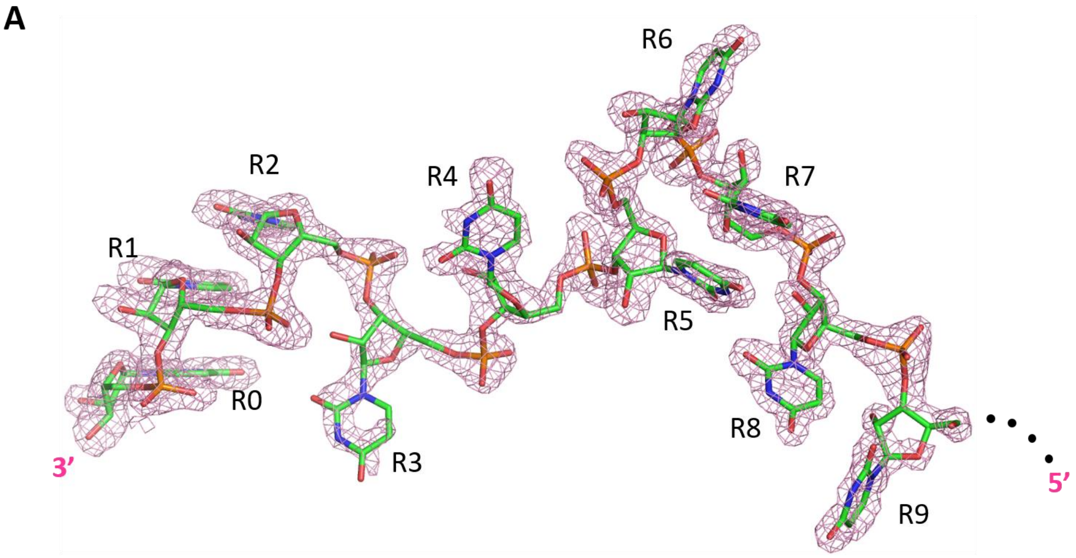

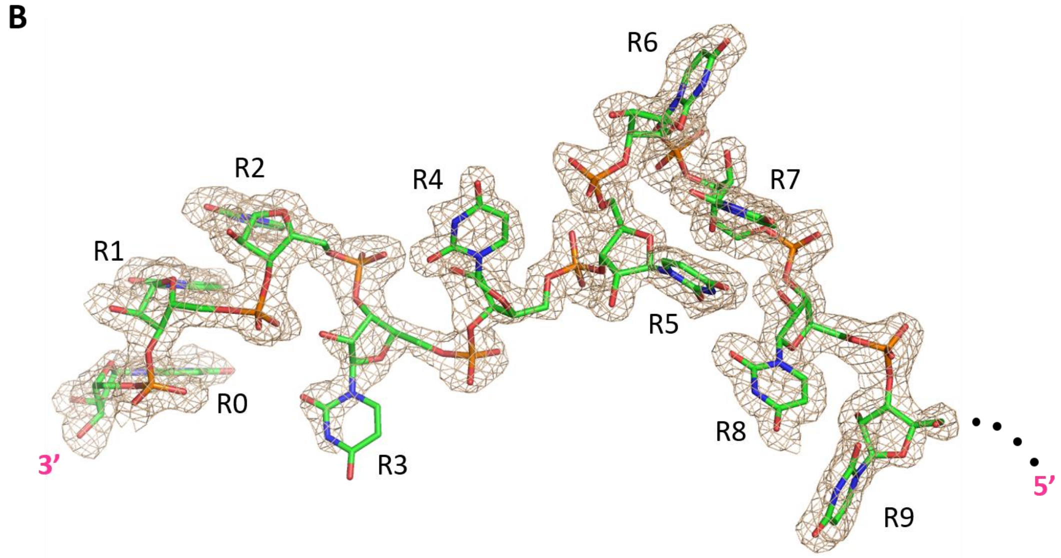

Fig. S4: Electron density maps of RNA-12U bound in the RNA-complexed structure.

(**A**) The $2F_o$-F_c simulated annealing omit maps. (**B**) The refined $2F_o$-F_c electron density maps. Both maps are contoured at 1.2 σ level.

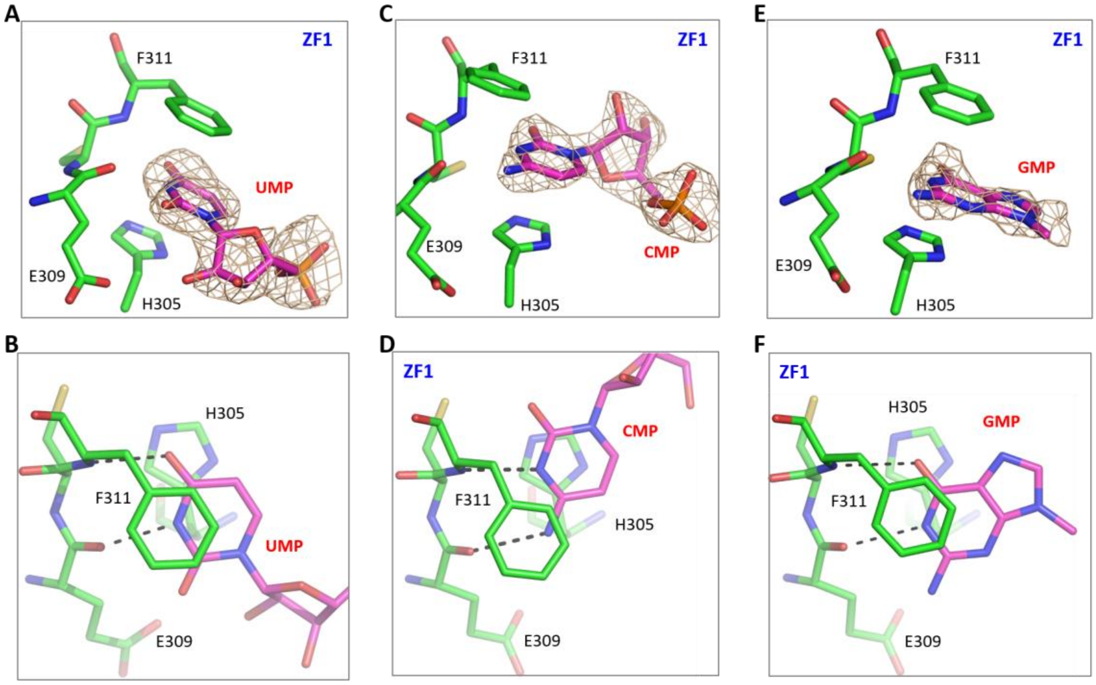

Fig. S5: NMPs captured in the ZF1 grooves of the NMP-bound structures.

(**A-B**) The $2F_o$-F_c simulated annealing omit maps and the interactions of UMP captured in the UMP-bound structure. (**C-D**) The $2F_o$-F_c simulated annealing omit maps and the interactions of CMP captured in the CMP-bound structure. (**E-F**) The $2F_o$-F_c simulated annealing omit maps and the interactions of GMP captured in the GMP-bound structure. The maps for UMP and CMP are contoured at 1.2 σ level, whereas they are contoured at 1.0 σ level for GMP.

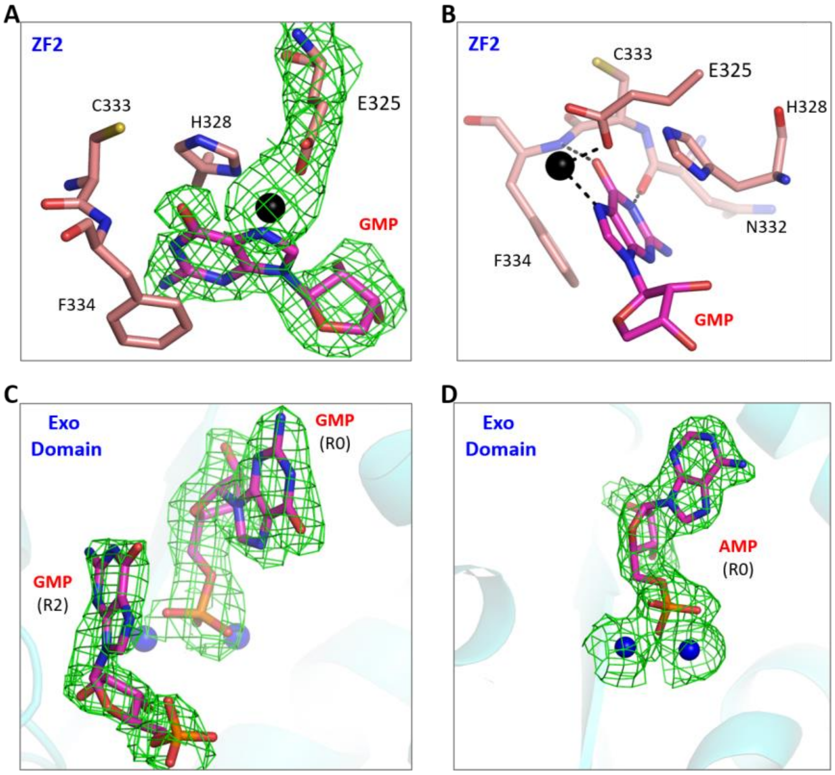

Fig. S6: NMPs captured in the ZF2 grooves or the active sites of the NMP-bound structures.

(**A-B**) The $2F_o$-F_c simulated annealing omit maps (contour level 1.0 σ) and the interactions of GMP captured in the ZF2 groove of the GMP-bound structure. (**C-D**) The $2F_o$-F_c simulated annealing omit maps (contour level 1.2 σ) of GMP and AMP captured in the active sites of the GMP-bound and AMP-bound structures, respectively. Mn^{2+} captured in the ZF2 groove and the active site are shown as spheres in black and blue, respectively.

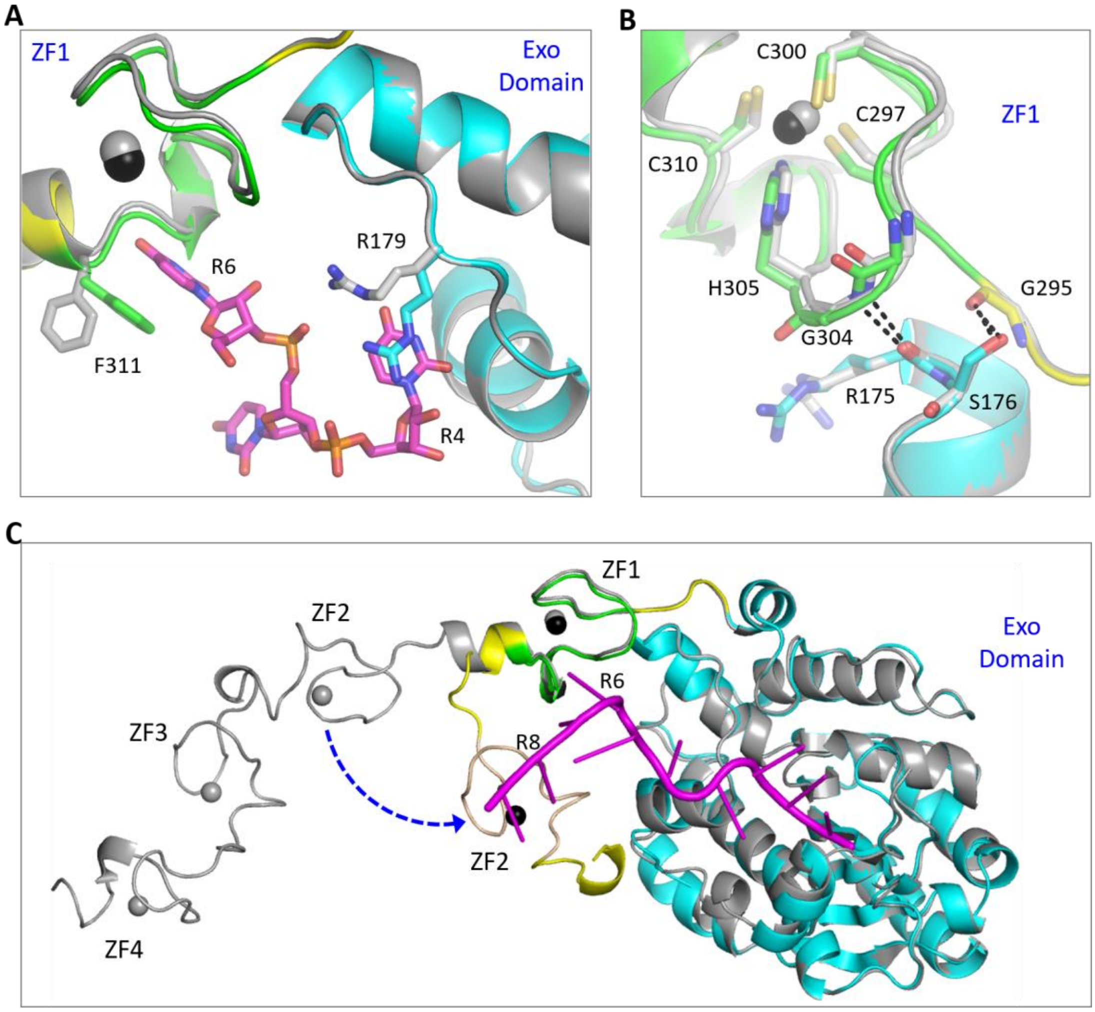

Fig. S7: Comparison of different *Tb*RND structures.

(**A**) Superposition of the apo- and RNA-complexed structures showing the conformation changes of the RNA-interacting residues. (**B**) Superposition of the apo- and RNA-complexed structures showing the relatively fixed orientation of the Exo domain and ZF1 motif of *Tb*RND. (**C**) Superposition of the GMP-bound and RNA-complexed structures showing the large conformational change for ZF2 motif. Both apo- and GMP-bound structures are colored in grey. The RNA-complexed structure is colored as in Fig. 2A.

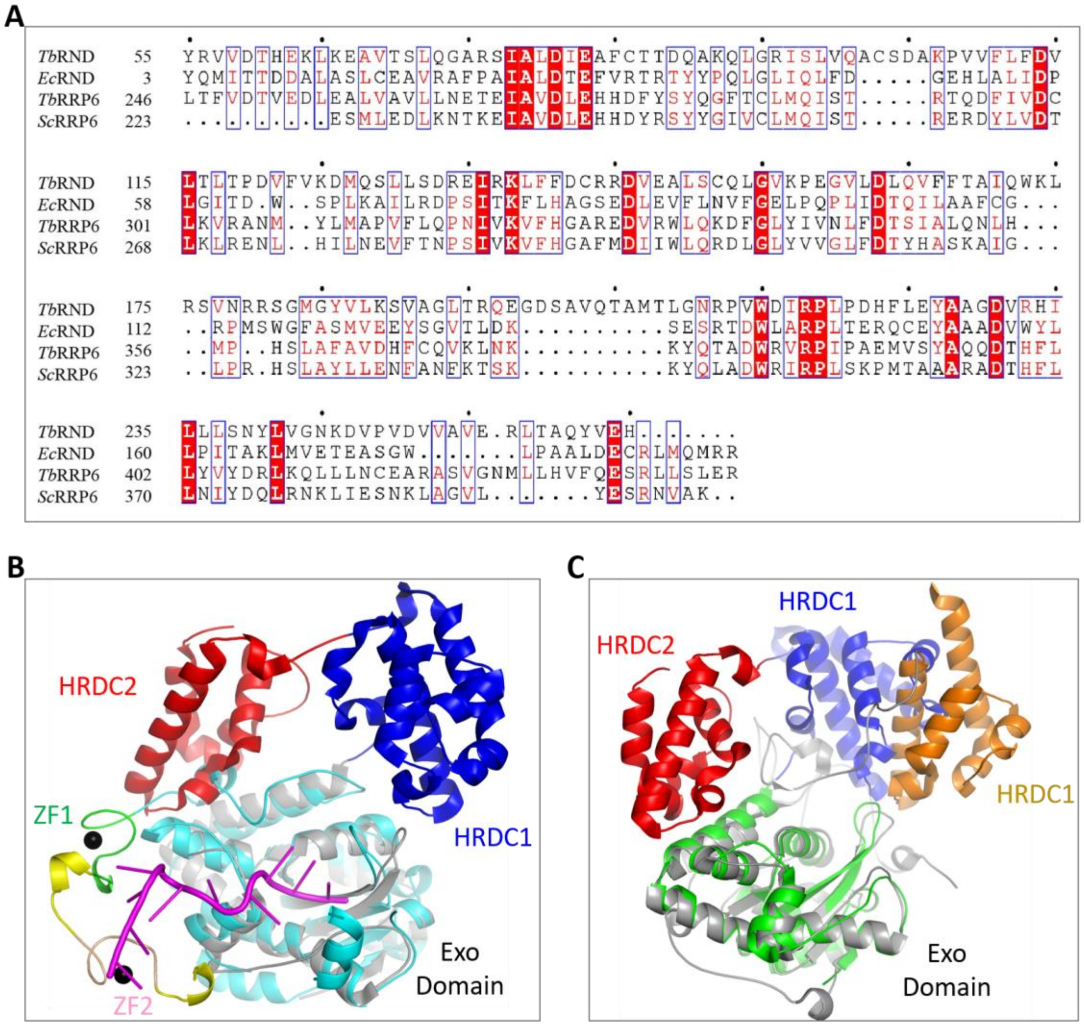

Fig. S8: Sequence alignment and structural comparison of RND family proteins. (**A**) Structure-based sequence alignment of the Exo domains of RND superfamily proteins. (**B**) Superposition of *Ec*RND and the RNA-complexed *Tb*RND structures. The RNA-complexed *Tb*RND structure is colored as in Fig. 2A. The Exo, HRDC1, and HRDC2 domains of *Ec*RND are colored in grey, blue, and red, respectively. (**C**) Superposition of *Ec*RND and *Sc*Rrp6 structures. *Sc*Rrp6 is colored in green and orange for the Exo and HRDC domains, respectively. *Ec*RND is colored as in (**B**).

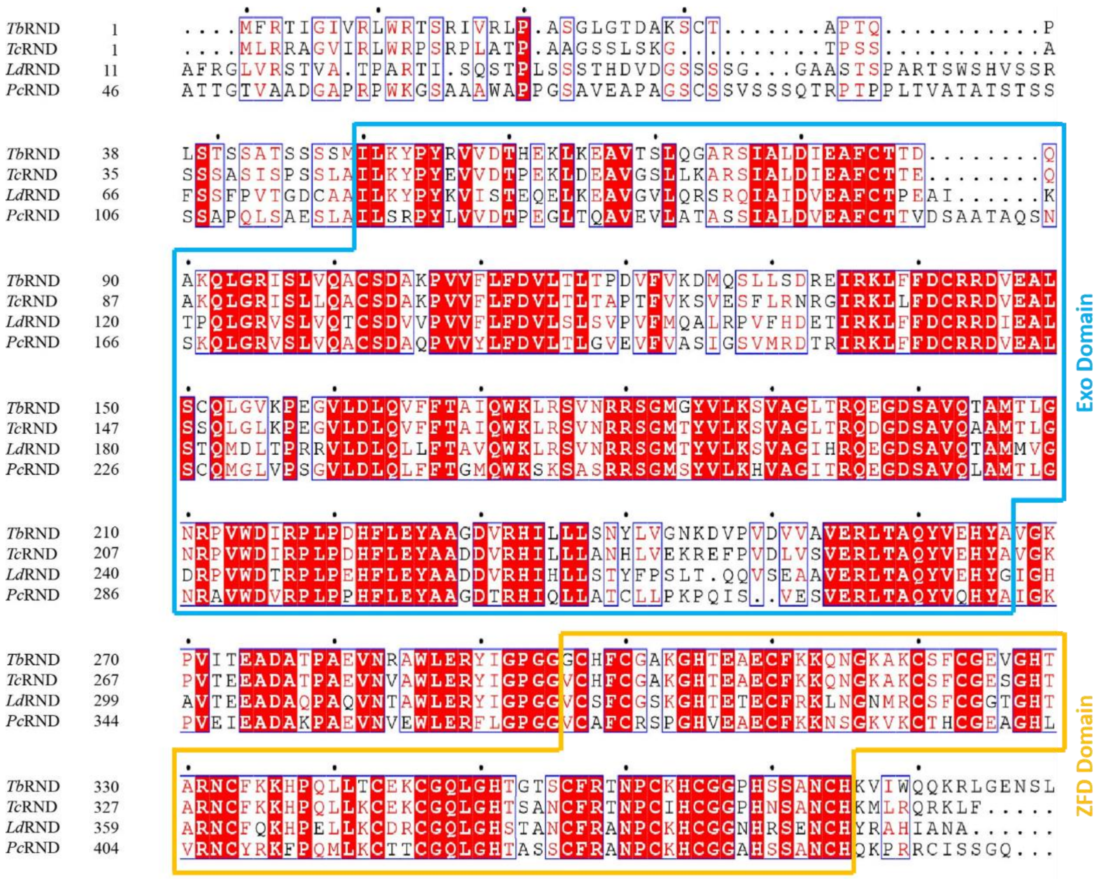

Fig. S9: Sequence alignment of trypanosomatid RND proteins. *Tb*, *Trypanosoma brucei*; *Tc*, *Trypanosoma cruzi*; *Ld*, *Leishmania donovani*; *Pc*, *Paratrypanosoma confusum*.

Table S1. Sequence of codon-optimized cDNA of *Tb*RND

```
5'-
ATGTTCCGTACCATCGGCATCGTGCGTCTCTGGCGCACCTCTCGTATCGTTCGTCT
CCCGGCGTCTGGTCTGGGCACCGACGCGAAATCTTGCACTGCGCCGACTCAGCCGC
TCTCTACCTCTAGCGCCACCTCTTCCTCTTCTATGATCCTGAAATACCCGTACCGT
GTCGTTGATACCCACGAAAAACTGAAAGAAGCGGTTACCTCTCTCCAGGGTGCGCG
TTCTATCGCACTGGACATCGAAGCCTTCTGCACGACCGACCAGGCGAAACAACTGG
GTCGCATCTCCCTCGTTCAGGCGTGTTCTGATGCCAAACCTGTTGTCTTTCTGTTC
GACGTTCTCACCCTCACCCCGGACGTTTTCGTTAAAGATATGCAGAGCCTCCTGTC
TGATCGTGAAATCCGTAAACTGTTCTTCGACTGCCGTCGTGATGTTGAAGCGCTGT
CTTGTCAGCTCGGTGTTAAACCAGAGGGTGTACTGGACCTGCAAGTTTTCTTCACT
GCGATCCAGTGGAAACTGCGTTCCGTTAACCGCCGTTCTGGCATGGGTTATGTGCT
GAAATCTGTAGCGGGTCTGACCCGTCAAGAGGGCGATAGCGCGGTTCAGACCGCGA
TGACCCTGGGTAATCGCCCAGTTTGGGACATTCGTCCACTGCCGGATCACTTCCTG
GAATACGCGGCTGGTGATGTACGTCATATCCTGCTGCTCAGCAACTATCTGGTTGG
TAACAAGGACGTACCGGTTGACGTTGTTGCGGTTGAACGTCTCACCGCACAATACG
TGGAACACTACGCCGTCGGTAAGCCTGTCATCACGGAGGCGGACGCGACCCCTGCG
GAGGTAAATCGCGCTTGGCTGGAACGTTACATCGGTCCAGGTGGTGGCTGTCATTT
CTGTGGCGCGAAAGGTCATACCGAAGCGGAATGTTTCAAGAAGCAAAACGGTAAAG
CGAAATGCTCCTTTTGCGGCGAGGTTGGCCACACCGCCCGTAACTGCTTCAAAAAG
CATCCGCAACTGCTCACCTGTGAAAAATGCGGTCAACTCGGTCACACGGGTACGAG
CTGCTTTCGCACCAACCCGTGCAAACATTGTGGCGGTCCGCACAGCTCCGCGAACT
GTCATAAAGTGATTTGGCAGCAGAAACGTCTGGGTGAAAACTCCCTCCACTAA
-3'
```

Table S2. Primers for truncated and mutant *Tb*RND construction.

Name	Sequence (from 5' to 3')
RND-WT_F	AAAGGATCCGGTGGTATGTCTAGCGCCACCTCT
RND-WT_R	AAACTCGAGTTAGTGGAGGGAGTTTTCACCC
ΔZF_3-4_R	AAACTCGAGTTAGAGCAGTTGCGGATGCTT
ΔZF_2-4_R	AAACTCGAGTTATTTACCGTTTTGCTTCTT
RND-Exo_R	AAACTCGAGTTAGCCACCACCTGGACCGAT
D80A_F	TCTATCGCACTGGCCATCGAAGCCTTCTGC
D80A_R	GCAGAAGGCTTCGATGGCCAGTGCGATAGA
E82A_F	GCACTGGACATCGCCGCCTTCTGCACGACC
E82A_R	GGTCGTGCAGAAGGCGGCGATGTCCAGTGC
D141E_F	AAACTGTTCTTCGAATGCCGTCGTGATGTT
D141E_R	AACATCACGACGGCATTCGAAGAACAGTTT
Q164A_F	GTACTGGACCTGGCCGTTTTCTTCACTGCG
Q164A_R	CGCAGTGAAGAAAACGGCCAGGTCCAGTAC
R179A_F	CGTTCCGTTAACGCCCGTTCTGGCATGGGT
R179A_R	ACCCATGCCAGAACGGGCGTTAACGGAACG
S181A_F	GTTAACCGCCGTGCCGGCATGGGTTATGTG
S181A_R	CACATAACCCATGCCGGCACGGCGGTTAAC
Y185A_F	TCTGGCATGGGTGCCGTGCTGAAATCTGTA
Y185A_R	TACAGATTTCAGCACGGCACCCATGCCAGA
D230A_F	TACGCGGCTGGTGCCGTACGTCATATCCTG
D230A_R	CAGGATATGACGTACGGCACCAGCCGCGTA
H305A_F	GGCGCGAAAGGTGCCACCGAAGCGGAATGT
H305A_R	ACATTCCGCTTCGGTGGCACCTTTCGCGCC
F311A_F	GAAGCGGAATGTGCCAAGAAGCAAAACGGT
F311A_R	ACCGTTTTGCTTCTTGGCACATTCCGCTTC
H328A_F	GGCGAGGTTGGCGCCACCGCCCGTAACTGC
H328A_R	GCAGTTACGGGCGGTGGCGCCAACCTCGCC
N332A_F	CACACCGCCCGTGCCTGCTTCAAAAAGCAT
N332A_R	ATGCTTTTTGAAGCAGGCACGGGCGGTGTG
F334A_F	GCCCGTAACTGCGCCAAAAAGCATCCGCAA
F334A_R	TTGCGGATGCTTTTTGGCGCAGTTACGGGC
H351A_F	GGTCAACTCGGTGCCACGGGTACGAGCTGC
H351A_R	GCAGCTCGTACCCGTGGCACCGAGTTGACC
H369A_F	TGTGGCGGTCCGGCCAGCTCCGCGAACTGT
H369A_R	ACAGTTCGCGGAGCTGGCCGGACCGCCACA

Table S3. Data collection and refinement statistics[a]

Structure<br><br>(PDB ID)	apo-form<br><br>7C42	GMP-bound (G-form)<br><br>7C4C	UMP-bound (U-form)<br><br>7C4B	CMP-bound (C-form)<br><br>7C47	AMP-bound (A-form)<br><br>7C43	ΔZF_3-4/RNA-12U complex<br><br>7C45
**Data collection**[a]						
Space group	$P2_12_12_1$	$P2_12_12_1$	$P2_12_12_1$	$P2_12_12_1$	$P2_12_12_1$	$P2_12_12_1$
Cell parameter:						
a (Å)	50.9	57.2	51.4	52.3	49.7	44.4
b (Å)	77.5	75.0	78.0	77.4	77.7	65.8
c (Å)	101.5	101.4	101.7	102.3	99.3	99.9
Wavelength (Å)	0.9793	0.9793	0.9793	0.9793	0.9793	0.9793
Resolution (Å)	30.0-2.0	30.0-2.25	30.0-2.1	30.0-2.2	30.0-2.3	50.0-1.77
Last shell (Å)	2.07-2.0	2.33-2.25	2.18-2.1	2.28-2.2	2.38-2.3	1.83-1.77
Completeness (%)	98.8(92.5)	98.3(92.5)	97.6(88.0)	98.6(91.5)	97.4(91.6)	95.2(90.2)
Redundancy	10.2(7.3)	7.3(3.6)	7.9(3.8)	9.5(6.4)	8.4(4.8)	4.1(3.9)
I/σ(I)	20.4(2.6)	18.3(2.3)	29.3(1.9)	32.4(3.4)	26.8(4.4)	15.0(2.0)
Rmerge (%)	11.6(62.2)	8.9(40.5)	9.0(51.8)	5.8(36.9)	7.1(27.9)	7.9(47.3)
**Refinement**						
Resolution (Å)	29.5-2.0	30.0-2.27	29.7-2.1	30.0-2.2	30.0-2.3	36.8-1.77
R_{work} (%) / R_{free} (%)	17.6/21.5	19.2/23.9	23.1/26.7	22.1/25.0	20.0/24.2	19.3/22.1
No. of atoms						
Protein	2271	2524	2239	2276	2292	2262
RNA	0	0	0	0	0	197
NMP	0	78	21	42	23	0
Cation	2	12	4	4	4	4
water	245	41	27	53	27	225
R.m.s. deviations						
Bond length (Å)	0.006	0.012	0.002	0.007	0.011	0.008
Bond angle (°)	0.763	0.940	0.482	1.086	1.534	0.989
Ramachandran plot (%)						
Most favored	97.6	95.9	98.0	98.0	98.0	95.5
Additional allowed	2.1	3.5	1.4	1.0	2.0	3.9
Outlier	0.3	0.6	0.6	1.0	0	0.6

[a]: Values in parentheses are for the last resolution shell.

Table S4. Sequences of RNAs used in crystallization and *in vitro* cleavage assays.

Name	Sequence (from 5' to 3')
RNA-12U	UUUUUUUUUUUU
RNA-12C	CCCCCCCCCCCC
RNA-12A	AAAAAAAAAAAA
RNA1	AACUUGUUUUUUUU
RNA1-R3C	AACUUGUUUU**C**UUU
RNA1-R3A	AACUUGUUUU**A**UUU
RNA1-R3G	AACUUGUUUU**G**UUU
RNA1-R4C	AACUUGUUU**C**UUUU
RNA1-R4A	AACUUGUUU**A**UUUU
RNA1-R4G	AACUUGUUU**G**UUUU
RNA1-R5C	AACUUGUU**C**UUUUU
RNA1-R5A	AACUUGUU**A**UUUUU
RNA1-R5G	AACUUGUU**G**UUUUU
RNA1-R6C	AACUUGU**C**UUUUUU
RNA1-R6A	AACUUGU**A**UUUUUU
RNA1-R6G	AACUUGU**G**UUUUUU
RNA2	AACUUGUUUUU**CAG**
RNA-5U	UUUUU
RNA-6U	UUUUUU
RNA-7U	UUUUUUU
gA6-14	GGACUAUAACUCCGAUAACGAAUCAGAUUUUGACAGUGAUAUGA<br>UAAUUAUUUUUUUUUUUUUUUU
A6U	GAGAAGAAAGGGAAAGUUGUGAUUUUGGAGUUAUAG

Table S5. Quantification of substrates and products at a reaction time of 30 min[a].

Protein	Substrate	R0 (%)	R4 (%)	R5 (%)	R4+R5 (%)
*Tb*RND	RNA1	ND	39.02	49.36	88.38
*Tb*RND_D141E	RNA1	ND	12.31	1.09	13.40
*Tb*RND_Q164A	RNA1	3.81	ND	ND	ND
*Tb*RND_R179A	RNA1	12.08	ND	ND	ND
*Tb*RND_S181A	RNA1	ND	32.51	40.74	73.25
*Tb*RND_Y185A	RNA1	ND	26.90	21.54	48.44
*Tb*RND_H305A	RNA1	13.09	5.04	ND	5.04
*Tb*RND_F311A	RNA1	12.21	4.85	ND	4.85
*Tb*RND_H328A	RNA1	ND	45.56	12.50	58.06
*Tb*RND_N332A	RNA1	ND	21.15	57.29	78.44
*Tb*RND_F334A	RNA1	ND	27.98	56.57	84.55
*Tb*RND_H351A	RNA1	ND	41.59	38.93	80.25
*Tb*RND_H369A	RNA1	ND	39.89	38.64	78.53
*Tb*RND ΔZF_2-4	RNA1	67.44	ND	ND	ND
*Tb*RND ΔZF_3-4	RNA1	ND	44.50	10.04	54.54

[a]: ND: not detected.

Table S6. Quantification of intact substrates at a reaction time of 1 min.

Protein	Substrate	R0 (%)
*Tb*RND	RNA1	13.08
*Tb*RND	RNA1-R3C	14.56
*Tb*RND	RNA1-R3A	37.17
*Tb*RND	RNA1-R3G	10.75
*Tb*RND	RNA1-R4C	13.80
*Tb*RND	RNA1-R4A	45.81
*Tb*RND	RNA1-R4G	44.98
*Tb*RND	RNA1-R5C	49.13
*Tb*RND	RNA1-R5A	49.02
*Tb*RND	RNA1-R5G	66.69
*Tb*RND	RNA1-R6C	86.87
*Tb*RND	RNA1-R6A	79.94
*Tb*RND	RNA1-R6G	54.29
